# Supplementary material for: Cryptic transmission of SARS-CoV-2 and the first COVID-19 wave
Source: Nature. 2021 Oct 25;600(7887):127–32. doi: 10.1038/s41586-021-04130-w (PMC8636257; doi:10.1038/s41586-021-04130-w)
Supplement: Supplementary file 1 — This file contains Supplementary Sections 1–9, Figs. 1–20, Tables 1–8 and References. [file 41586_2021_4130_MOESM1_ESM.pdf]

nature portfolio

https://doi.org/10.1038/s41586-021-04130-w

Supplementary information

Cryptic transmission of SARS-CoV-2 and the first COVID-19 wave

In the format provided by the authors and unedited

Supplementary Information: Cryptic transmission of SARS-CoV-2 and the first COVID-19 wave

Jessica T. Davis[*,a], Matteo Chinazzi[*,a], Nicola Perra[*,b,a], Kunpeng Mu[a], Ana Pastore y Piontti[a], Marco Ajelli[c,a], Natalie E. Dean[d], Corrado Gioannini[e], Maria Litvinova[c], Stefano Merler[f], Luca Rossi[e], Kaiyuan Sun[g], Xinyue Xiong[a], Ira M. Longini Jr.[h], M. Elizabeth Halloran[i,j], Cécile Viboud[g], and Alessandro Vespignani[†a]

[a]*Laboratory for the Modeling of Biological and Socio-technical Systems, Northeastern University, Boston, MA USA*

[b]*Networks and Urban Systems Centre, University of Greenwich, London, UK*

[c]*Department of Epidemiology and Biostatistics, Indiana University School of Public Health, Bloomington, IN, USA*

[d]*Department of Biostatistics and Bioinformatics, Emory University, Atlanta, USA*

[e]*ISI Foundation, Turin, Italy*

[f]*Bruno Kessler Foundation, Trento Italy*

[g]*Division of International Epidemiology and Population Studies, Fogarty International Center, National Institutes of Health, Bethesda, MD, USA*

[h]*Department of Biostatistics, College of Public Health and Health Professions, University of Florida, Gainesville, USA*

[i]*Fred Hutchinson Cancer Research Center, Seattle, WA, USA*

[j]*Department of Biostatistics, University of Washington, Seattle, WA, USA*

October 6, 2021

Contents

1 Model Description **3**
1.1 Global Epidemic and Mobility Model . . . 3
1.2 Interventions Timeline . . . 6

2 Model Calibration **8**

3 Sensitivity Analysis **15**
3.1 Unconstrained pandemic evolution realizations . . . 15
3.2 Alternative distance measure for model calibration . . . 15
3.3 Overdispersion of disease transmission . . . 18

4 SARS-CoV-2 Introduction Statistics **22**

5 SARS-CoV-2 Seeding Networks **25**

6 Correlation Analysis **28**

7 Analysis of additional countries in different regions of the world **32**
7.1 Onset of local transmission . . . 32
7.2 COVID-19 burden . . . 32

*These authors contributed equally to this work.
†To whom correspondence should be addressed; E-mail: a.vespignani@northeastern.edu.

8 Counterfactual scenarios for testing and infection detection **36**

9 Data **38**
9.1 Epidemic surveillance data 38
9.2 Model intervention data 38
9.3 Serological data comparison 38

1 Model Description

1.1 Global Epidemic and Mobility Model. We use the Global Epidemic and Mobility model (GLEAM), a stochastic, spatial, epidemic model based on an age-structured, metapopulation approach that has been used and published previously (1; 2). In the model, the world is divided into over 3,200 geographic subpopulations constructed using a Voronoi tessellation of the Earth's surface. Subpopulations, centered around major transportation hubs (e.g., airports), consist of cells with a resolution of 15 x 15 arc minutes (approximately 25 x 25 kilometers). High resolution data are used to define the population of each cell (3). Other attributes of individual subpopulations, such as age specific contact patterns, health infrastructure, etc., are added according to available data (4; 5).

GLEAM integrates a human mobility layer, represented as a network, using both short-range (i.e., commuting) and long-range (i.e., flights) mobility data from the Offices of Statistics for 30 countries on 5 continents as well as the Official Aviation Guide (OAG) and IATA databases (updated in 2019) (6; 7). The air travel network of origin-destination travel (i.e.,integrating connecting flight information) consists of the daily passenger flows between airports, worldwide, mapped to their corresponding subpopulations. We define a worldwide homogeneous standard for GLEAM to overcome differences in the spatial resolution of the commuting data across different countries. Where information is not available, the short-range mobility layer is generated synthetically by relying on the "gravity law" or the more recent "radiation law" both calibrated using real data available (8). These approaches assume more frequent travel to nearby or closer subpopulations and less frequent travel to distant locations. In Fig. S1 we show a representation of the geographical resolution of the model for a few selected regions, both the long range and short range mobility networks, and the population structure at the global level.

Initial conditions are set specifying the number and location of individuals capable of transmitting the infection. GLEAM is then able to track over time the proportion of the population in each disease compartment for all subpopulations. At the start of each simulated day, travelers move to their destinations via the flight network. The probability of air travel changes from day to day, varies by age group, and can consider the effects of location specific airline traffic reductions. Short-range mobility (i.e., commuting) varies by disease status. Each full day is simulated using 12 distinct time steps, and this process is repeated for every simulated day. Individuals and their traveling patterns are tracked as shown in the flow diagram for the GLEAM algorithm (Fig. S2).

The combined population structure and mobility network create a synthetic world that is used to simulate the unfolding dynamics of the epidemic. The infection dynamics occur within each subpopulation. We adopt a classic $SLIR$ model in which individuals are classified into four compartments: susceptible, latent, infectious, or removed. Susceptible individuals become latent through interactions with infectious individuals. Latent individuals progress to the infectious stage at a rate inversely proportional to the latent period, and infectious individuals progress to the removed stage at a rate inversely proportional to the infectious period. During both the latent and infectious stages we assume that individuals are able to travel. Following the infectious period, individuals then progress into the removed compartment where they are no longer able to infect others, meaning they have either recovered, been hospitalized, or isolated. Individuals transition between compartments using stochastic binomial chain processes assuming parameter values from available literature that define the natural history of infection. In Table. S1 we report the parameter estimates used in the model. We estimate the number of deaths using the number of individuals in the removed compartment and assume the infection fatality ratio (IFR) has a uniform prior from $0.4\% - 2\%$ and is age-stratified proportional to the values reported in Verity et al. (9).

Once the mobility data layers and the disease dynamics are defined, the number of individuals in each compartment m, age bracket i, and subpopulation j follows a discrete and stochastic dynamical equation that reads as

$$X_j^{[m,i]}(t+\Delta t) - X_j^{[m,i]}(t) = \Delta X_j^{[m,i]} + \Omega_j([m,i]) \tag{1}$$

where the term, $\Delta X_j^{[m,i]}$, represents the change due to the compartment transitions induced by the

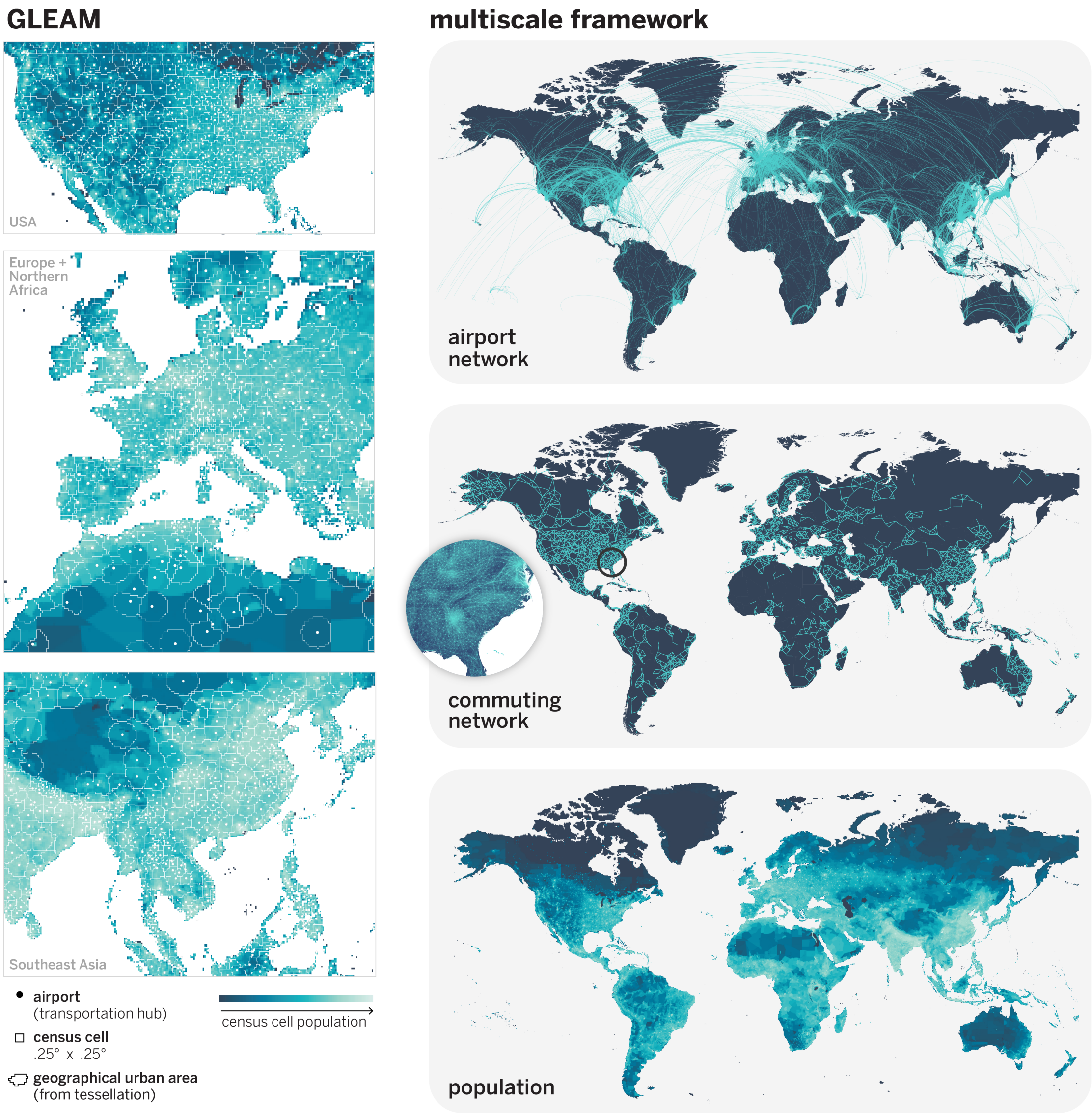

Figure S1: Schematic representation of GLEAM. (left) The subpopulation structure for selected regions. Subpopulations are geographic regions, formed from the Voronoi tessellation centered around airports. They are comprised of census cells that are approximately 25km x 25km. (right) Diagrams of the multiple mobility networks and population layer (from top to bottom): (1) the origin-destination airport network (long range mobility network), (2) the commuting network (short-range mobility network), (3) the population layer showing the population size of census cells.

disease dynamics and the transport operator, $\Omega_j([m,i])$, represents the variations due to the traveling and mobility of individuals. The latter operator takes into account the long-range airline mobility and defines the minimal time scale of integration as 1 day. The mobility due to the commuting flows is taken into account by defining effective force of infections by using a time scale separation approximation as detailed in Ref. (1). The $\Delta X_j^{[m,i]}$ is defined as the sum over all of the transitions into and out of disease compartment m of individuals in age group i ($[m,i]$). The operator $\mathcal{D}_j([m,i],[n,i])$ represents the number of transitions from $[m,i]$ to $[n,i]$ during the time interval Δt and each element of this operator is a random variable extracted from a multinomial distribution. The change $\Delta X_j^{[m,i]}$ of a compartment $[m,i]$ in this

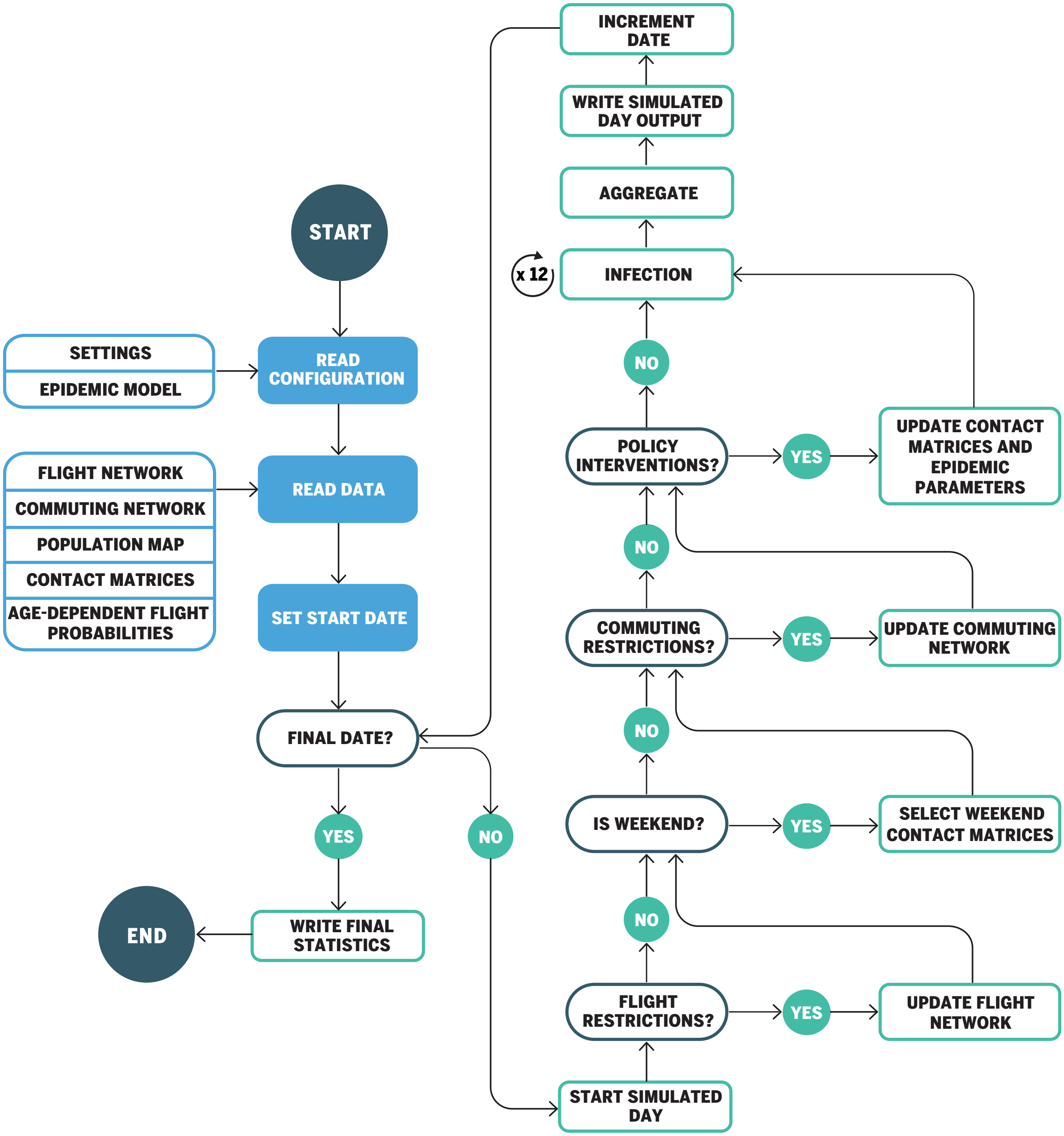

Figure S2: Flow diagram of GLEAM's algorithm

Parameters	Range	Ref.
Latent period (mean)	[4, 7] days	(10)
Infectious period (mean)	[2, 4] days	(11)
Days until recovery	[10, 14] days	(11; 9)
Generation time	[6, 8] days	(12; 13)

Table S1: Summary of parameter ranges explored in the sensitivity analysis. Reference parameters are reported in the main text

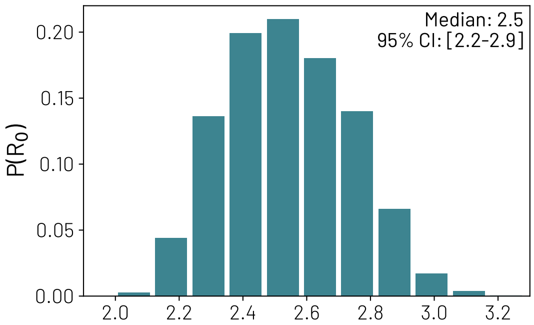

Figure S3: Posterior distribution of the reproductive number in China in the absence of mitigation policies.

time interval is given by a sum over all random variables $\{\mathcal{D}_j([m,i],[n,i])\}$ as follows

$$\Delta X_j^{[m,i]} = \sum_{[n,i]} \{-\mathcal{D}_j([m,i],[n,i]) + \mathcal{D}_j([n,i],[m,i])\}\,. \tag{2}$$

As a concrete example let us consider the evolution of the latent compartment. Individuals in age group i of subpopulation j can either transition into the Latent compartment (L_j^i) from the susceptible compartment (S_j^i) or transition out the Latent compartment into Infectious (I_j^i). The elements of the operator acting on L_j^i, are extracted from the binomial distributions: $Pr^{Bin}(L_j^i(t), p_{L_j^i \to I_j^i})$ and $Pr^{Bin}(S_j^i(t), p_{S_j^i \to L_j^i})$, where $p_{L_j^i \to I_j^i}$ and $p_{S_j^i \to L_j^i}$ are the transition probabilities from the latent state to the infectious state and from susceptible to the latent state, respectively. We assume a memoryless, discrete, stochastic transition processes. The probability $p_{S_j^i \to L_j^i}$ is the force of infection and it is determined by commuting flows, pattern of interactions as encoded in the age structured contact patterns, and the local non-pharmaceutical interventions (NPIs). We consider individuals divided into 10 age groups: [0-9, 10-19, 20-24, 25-29, 30-39, 40-49, 50-59, 60-69, 70-79, 80+]. The contacts matrix $\mathbf{C}$ considers interactions in four specific social settings: contacts at school ($\mathbf{C}_{school}$), workplace ($\mathbf{C}_{work}$), home ($\mathbf{C}_{home}$), and in the general community ($\mathbf{C}_{community}$). Therefore, in general the contacts matrix is a linear combination of the four contributions according to the contact reductions in different locations $\mathbf{C} = \sum_s \omega_s \mathbf{C}_s$, where ω_s indicates the number of contacts per setting, and s indicates the different settings mentioned before. The baseline ω_s and $\mathbf{C}_s$ values for each specific country are from Ref. (4). *For the sake of space we refer the reader to Ref. (1) where the analytical framework used in the model is reported in detail.* It is worth mentioning that the offspring distribution of infectious individuals will depend on the specific details of each population, local and global mobility, NPIs etc. A full discussion of the model overdispersion for secondary infections and a specific sensitivity analysis is provided in Section 3.3.

1.2 Interventions Timeline. In order to realistically depict the evolution of the epidemic, a comprehensive set of policy interventions is applied to modify disease transmissibility and population mobility. On January 15, partial international travel reductions (from 10% to 40%) are applied for individuals traveling to/from China (14). Between January 23 and 28, flight and commuting reductions are applied to Wuhan and other subpopulations in the Hubei province to enforce government-mandated quarantines.

In addition, on January 25, commuting reductions are applied also to all other subpopulations in mainland China. To do so, we collected daily travel data starting January 1, 2020 until February 25, 2020 from the Baidu Qianxi platform (15), which provides three mobility indices (i.e., inflow index, outflow index, and intra-city index). The indices are proxies for the number of travelers moving in, out of, and inside a city, respectively. We extracted the mobility outflow index of 27 provinces and 4

municipalities for the year 2020 and the previous year (with the same lunar date), and then mapped all provinces and municipalities to the metapopulation structure of the model to estimate the travel flow changes during the epidemic where the travel reduction can be estimated as $1 - \frac{I_{cur}}{I_{pre}}$, where I_{cur} and I_{pre} are the mobility outflow index of year 2020 and previous year on the same lunar date, respectively.

On February 1, due to the increasing amount of restrictions implemented by various countries and airlines (16; 17; 18; 19; 20; 21), stronger travel reductions are applied between mainland China and the rest of the world. We use actual worldwide (both international and domestic) origin-destination traffic data from the OAG database to quantify travel reductions. We also apply case detection based on travel history and additional travel bans across pairs of countries according to the Oxford COVID-19 Government Response Tracker (OxCGRT) (22). We account as well for the intra-country mobility and contacts reduction in workplaces and social settings (23) using the COVID-19 Community Mobility reports obtained from Google (24).

From mid-March 2020 all around the world, countries started to close schools as a means to slow the spread of COVID-19. We use the timeline of school closures provided by OxCGRT (22). As our model considers contact matrices for different settings, namely households, schools, workplaces and community contacts (4; 5), we quantify the decrease in contacts that individuals have in each of these environments. To implement school closures in the United States and the rest of the countries we follow (25) where authors study the effects of school closure in the context of seasonal influenza epidemics. According to the date when schools were closed in the different states/countries we consider a reduction of contacts in all individuals attending an educational institution (22). In the United States, Spain, and Italy, this intervention was applied at state/region level and for the rest of the European countries analyzed it was applied at country level.

Following school closures, most US states and European countries issued *stay-at-home* orders. In this case, we consider that contacts occurred exclusively in the household and essential workplaces. Using the COVID-19 Community Mobility reports (24) we compute the relative reduction in the number of contacts in workplaces and community interactions as well as the relative reduction in the intra-country mobility. We used data at the state or regional level for the United States, Italy, and Spain starting on February 15, 2020 and at the country level for all other countries available. For countries where we do not have mobility reports available we assume that on the date that schools closed there is a reduction in mobility of 50%, and an 100% reduction when there is a *stay-at-home* order. When the interventions are relaxed the mobility reduction is relaxed accordingly.

From the Google mobility reports we use the field `workplaces percent change from baseline` to infer contacts reductions in workplaces and the field `retail and recreation percent change from baseline` to infer contacts reductions in the general community setting. The Google mobility report provides the percentage change $r_l(t)$ on day t of total visitors to specific locations s with respect to a pre-pandemic baseline calculated as the median value, for the corresponding day of the week, during a 5 weeks period from January 3 until February 6, 2020. We turn this quantity into a rescaling factor for contacts such as $\omega_s(t) = \omega_s(1 + r_l(t)/100)^2$, by considering that the number of potential contacts per location scales as the square of the the number of visitors. We also use the ordinal index `C1 School closing` from the Oxford Coronavirus Government Response Tracker to modulate contacts in schools and universities. The index ranges from a minimum of 0 (no measures) to a maximum of 3 (require closing all levels). Furthermore, all ω factors are multiplied (or set equal to in case of contacts at home) by setting-specific weights from Mistry et al. (4). Finally we explore different level of overall transmissibility reduction (0-30%, step 10%) due to the awareness of population and behavioral changes starting at the date of the state of the emergency in the US and EU countries.

2 Model Calibration

The model described is stochastic and outputs an ensemble of possible epidemic outcomes for each set of initial conditions. We seed the epidemic in Wuhan, China assuming a starting date between November 15 and December 1, 2019, with 20 initial infections (26). Given the doubling time of the epidemic, this might corresponds to the virus emerging in mid October to late November, 2019 (27; 28; 29; 30; 26). We simulate more than 200,000 global epidemic realizations sampling reproductive numbers (R_0) from a uniform prior in the range 1.6 to 3.3 (step 0.01). We use an Approximate Bayesian Computation (ABC) Rejection Algorithm to sample a set of parameter points θ (for instance R_0) according to a prior distribution and simulates through the model the dataset E'. A distance measure $s(E', E)$ determines the difference between E' and the evidence E based on a given metric. If the generated E' is outside a tolerance from the evidence E (i.e., $s(E', E) > \epsilon$) the sampled parameter value is discarded. The sampled parameters that are accepted provide an estimate of the likelihood with respect to the evidence E and allows us to calculate the posterior distribution $P(\theta, E)$. As evidence, E, we considered the cumulative number of SARS-CoV-2 cases internationally imported from China up to January 21, 2020. The distance measure at each date is the difference between the SARS-CoV-2 cumulative imported cases generated by the model and the evidence with a tolerance provided by the under-detection interval estimated in Ref. (31). More specifically, only a fraction of imported cases are detected at the destination (32). According to the estimates proposed in Ref. (33), we stratify the detection capacity of countries relative to Singapore into three groups: high, medium and low surveillance capacity according to the Global Health Security Index (34), and assume an overall detection capacity for Singapore varying from 30% to 100% of imported cases. We also account for a non detectable 40% rate of asymptomatic individuals (sensitivity analysis ranging from 35% to 50%) (35; 36). The rejection algorithm accepts only configurations that satisfy the distance measure every day considered in the above time interval. This approach allows us to calibrate the model by incorporating both the growth rate of importations and their magnitude, scaled according to the under-detection estimates. The detailed list of importation events used is provided in Table S1 of the supplementary materials of Ref. (14). Using the ABC calibration and the age-stratified contact matrices, the obtained posterior distribution $P(R_0 = x|E)$ for the basic reproductive number R_0 in China has a median of 2.5 [95% CI 2.2-2.9] (Fig. S3), with a median doubling time of 3.8 [95% CI $3.1 - 4.6$] days in the absence of mitigation policies, for an overall detection capacity in Singapore of 60%. The posterior of R_0 in China has small variations, yielding a median of 2.4 [95% CI 2.1-2.8] and 2.7 [95% CI 2.3-3.1] for an overall detection capacity in Singapore of 100% and 30% respectively. In Fig. S4 we show the posterior distributions of the onset of local transmission for the 49 continental United States and 30 European countries investigated using this calibration.

To estimate the posterior distribution of the IFR and infection attack rate (AR) in each US state and European country, we use an additional ABC rejection approach comparing the weekly model-estimated deaths with the reported ones. Specifically, we consider the subset of realizations that are (i) consistent with the international importations from China up to January 21, 2020 (i.e., selected from the global model calibration) and (ii) show Italy as the first country, in the group under examination, to experience sustained local transmission (more details in section 3A). Then we estimate, for each realization in each state and country considered, the number of deaths from the removed compartment by considering an uniformly distributed IFR prior ranging from 0.4% to 2% that is age stratified proportionally to the values estimated by Ref. (9). We also consider that deaths are subject to a reporting delay uniformly distributed between $2 - 22$ days for both the US and Europe. As a distance measure, $s(E', E)$, for the ABC rejection algorithm we use the summary statistics provided by the the weighted mean absolute percentage error ($wMAPE$):

$$wMAPE = \frac{\sum_t |D_{model}(t) - D_{reported}(t)|}{\sum_t D_{reported}(t)} * 100$$

where D_{model} corresponds to the delayed/shifted model-estimated deaths and $D_{reported}$ to the reported

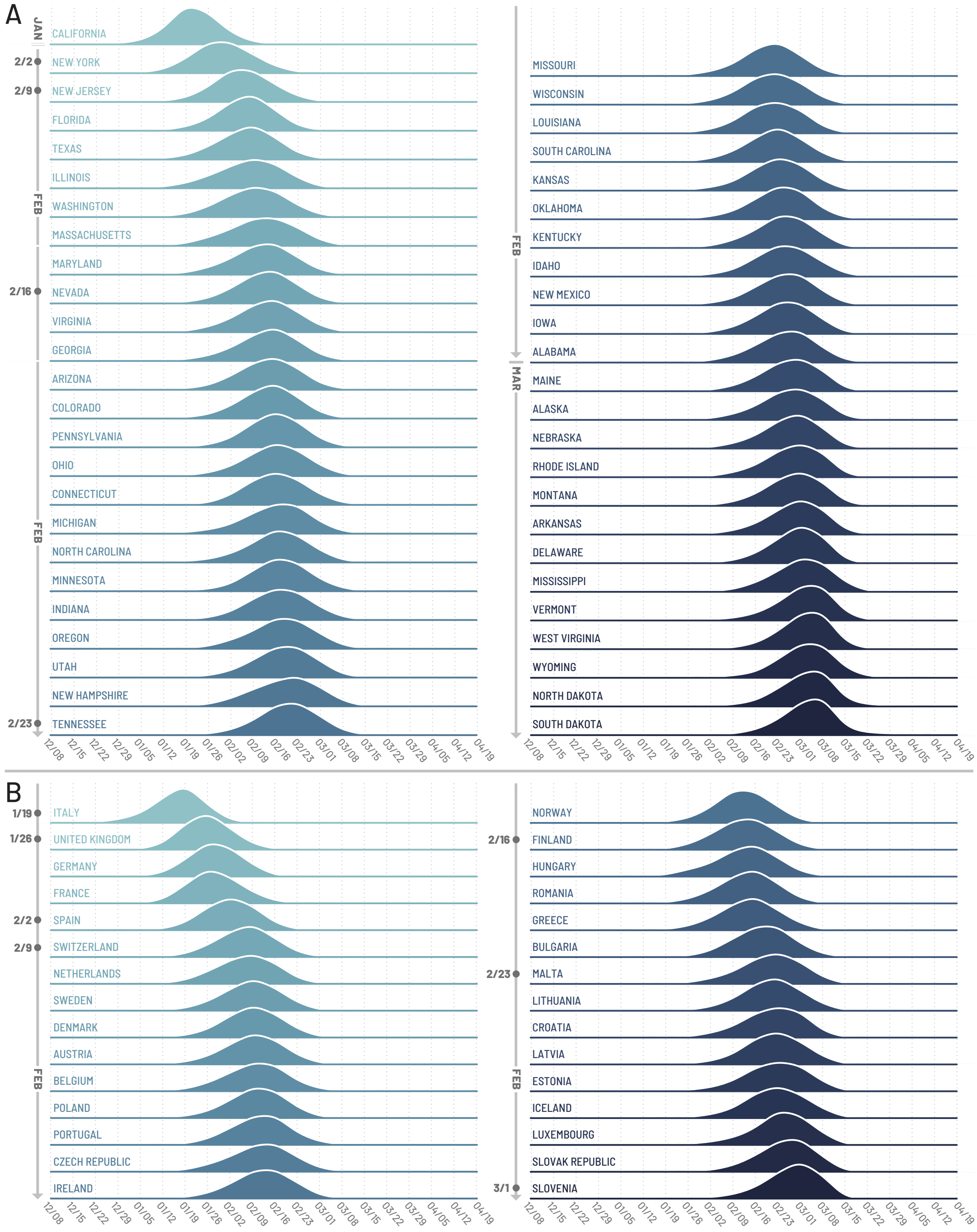

Figure S4: **Timing of the onset of local transmission for all countries and US states investigated.** We plot the posterior distributions of the week when each US state (A) or European country (B) first reached 10 locally generated SARS-CoV-2 transmission events per day. Countries/states are ordered by the median date of their posterior distribution. The week of this date corresponds to the dates reported on the the vertical axis.

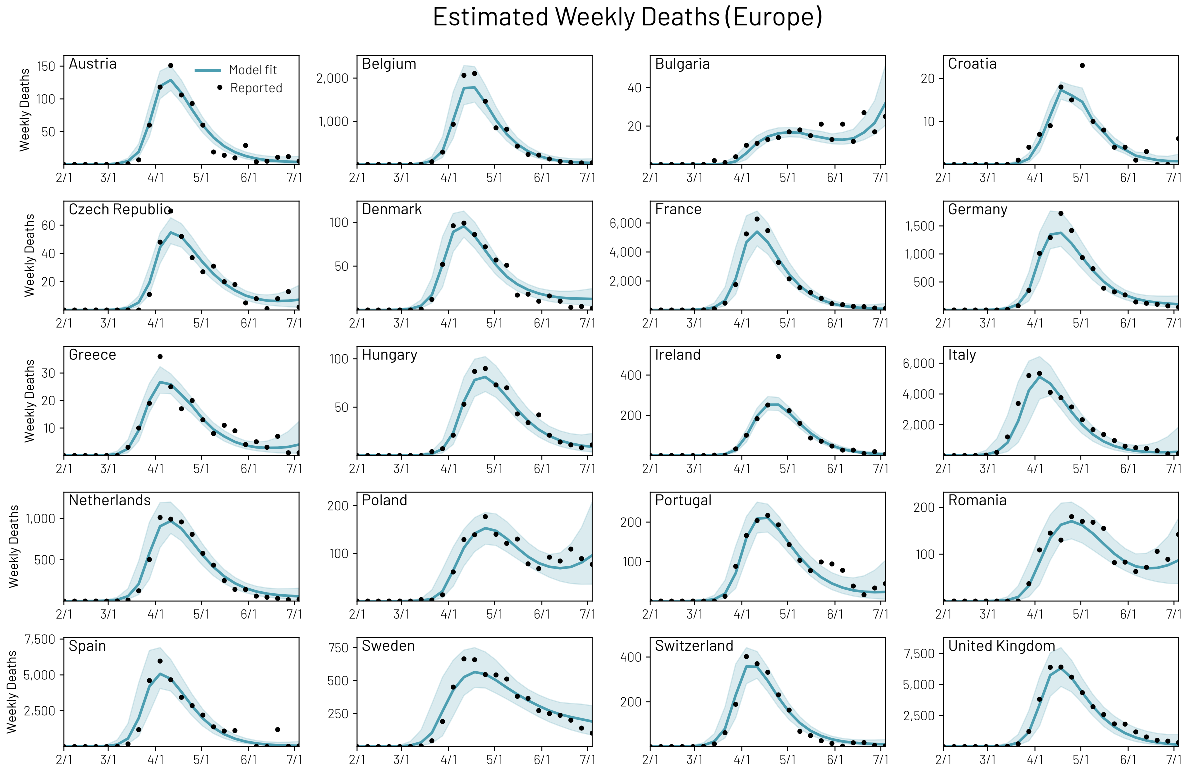

Figure S5: Estimated weekly deaths for 20 European countries using the wMAPE state/country level calibration method with a 25% tolerance threshold. We report the median value and the 90% confidence interval.

data. We only consider the deaths that were reported between March 22, 2020 and June 27, 2020, and set a tolerance of 25%, keeping only the realizations with a $s(E', E) = wMAPE < 25\%$. Using this approach we generate estimates and credible intervals for the infection AR and IFRs for 36 US states and 20 European countries. We extend to a larger set of countries in a later section of the SI. In the main text we show the model fits of the weekly deaths for four US states and four European countries. In Fig. S5 and Fig. S6 we show the estimated weekly deaths with the reported values for all calibrated European countries and US states. We also include Tables S2 and S3 which report the infection AR, IFR, and reproductive number (R_0) for each US state and European country, respectively.

In Fig. S7 we show the correlation between the weekly estimated deaths and the reported values from surveillance data. This is to provide a test of the goodness of fit attained by the wMAPE ABC calibration as in our approach the rejection criteria is the wMAPE on the whole epidemic profile and not on the individual weekly values. We find a Pearson correlation coefficient of 0.99 ($p < 0.001$) from the results for both the US states and European countries. It is also important to stress that the calibration on weekly reported deaths is subject to the bias' of that data, for example: under-reporting, the use different definitions of COVID-19 deaths (e.g., some states/countries report both probable and confirmed deaths while others only report confirmed deaths), and outliers that are a result of states/countries reporting backlogged data on a single day.

Additionally, to analyze the stability of our selected list of states/countries from the calibration reported in the main text, we tested different tolerances of the $wMAPE$ scores. Increasing the tolerance to 30% adds 4 US states and 2 European countries (US: Utah, Maine, Colorado, Iowa; EU: Slovenia and Norway). Decreasing the tolerance to 20% removes 5 US states and 3 European countries (US: Mississippi, Nebraska, New Hampshire, Oregon, Wisconsin; EU: Greece, Croatia, Bulgaria). However, results using the different tolerance values do not deviate with respect to what was reported in the main

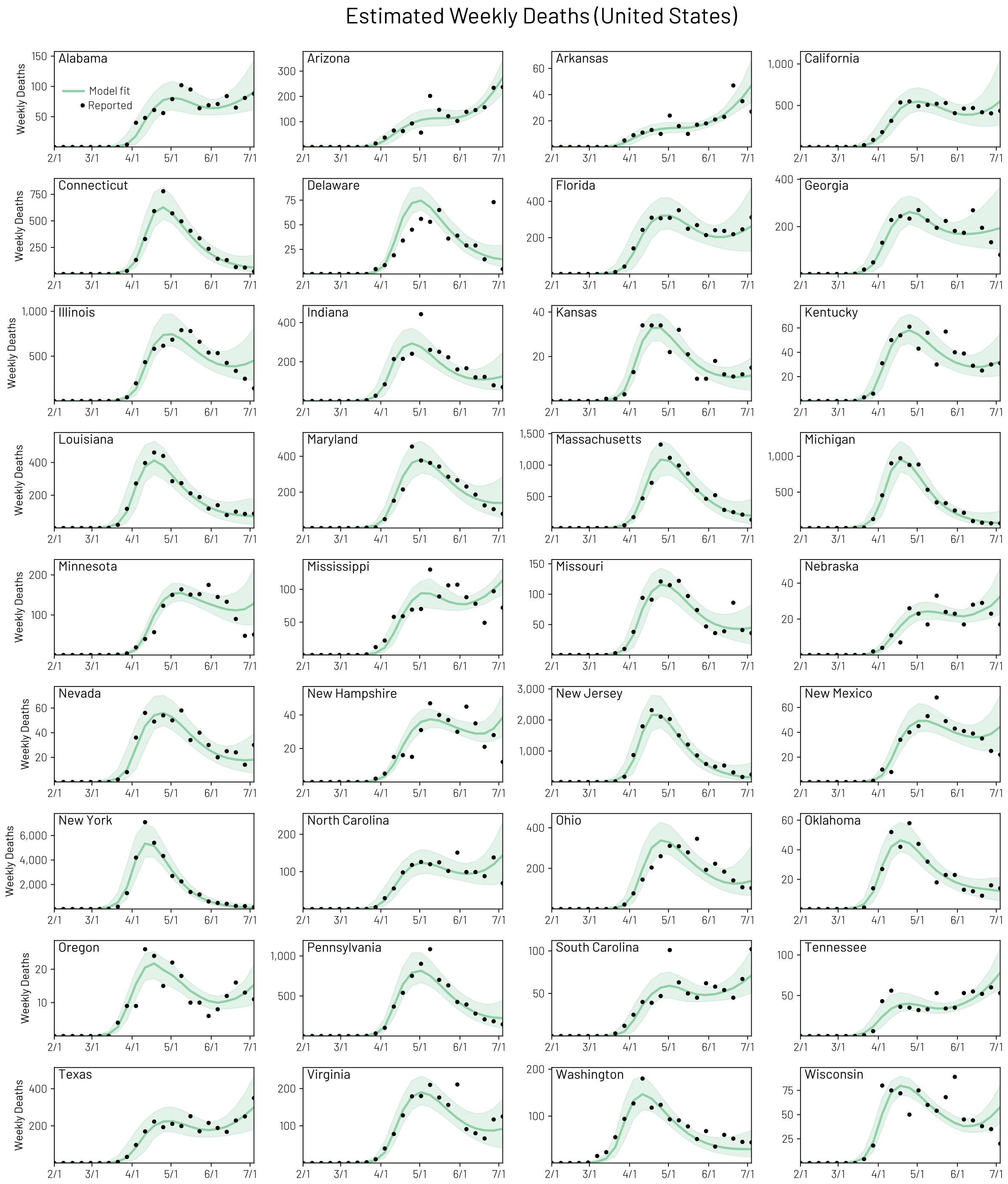

Figure S6: Estimated weekly deaths for 36 US states using the wMAPE state/country level calibration method with a 25% tolerance threshold. We report the median value and the 90% CI.

**Name**	Infection Attack Rate (%)	IFR (%)	R_0
Austria	1.16 [0.74, 2.85]	0.81 [0.35, 1.23]	2.61 [2.33, 2.83]
Belgium	13.24 [8.50, 28.35]	0.71 [0.33, 1.00]	2.73 [2.34, 2.99]
Bulgaria	0.98 [0.48, 2.35]	1.06 [0.38, 1.63]	2.66 [2.26, 2.80]
Croatia	0.19 [0.12, 0.42]	1.33 [0.57, 2.04]	2.47 [2.24, 2.69]
Czech Republic	0.46 [0.27, 1.22]	0.86 [0.37, 1.31]	2.59 [2.32, 2.83]
Denmark	1.29 [0.82, 3.16]	1.00 [0.41, 1.43]	2.50 [2.24, 2.69]
France	4.79 [3.38, 10.31]	1.01 [0.47, 1.31]	2.78 [2.47, 3.02]
Germany	1.16 [0.73, 2.89]	1.02 [0.42, 1.47]	2.59 [2.34, 2.81]
Greece	0.19 [0.10, 0.46]	0.99 [0.40, 1.66]	2.58 [2.34, 2.83]
Hungary	0.80 [0.48, 2.02]	0.87 [0.35, 1.27]	2.62 [2.34, 2.85]
Ireland	5.04 [3.20, 12.19]	0.71 [0.30, 1.05]	2.73 [2.41, 2.96]
Italy	4.51 [3.13, 10.83]	1.37 [0.63, 1.78]	2.76 [2.38, 3.01]
Netherlands	4.96 [3.13, 11.65]	0.85 [0.37, 1.28]	2.69 [2.37, 2.93]
Poland	0.60 [0.30, 1.46]	0.94 [0.39, 1.56]	2.57 [2.32, 2.80]
Portugal	1.56 [0.94, 3.67]	1.07 [0.45, 1.53]	2.69 [2.38, 2.92]
Romania	1.07 [0.64, 2.53]	0.94 [0.39, 1.33]	2.69 [2.38, 2.95]
Spain	7.30 [5.18, 14.79]	1.09 [0.55, 1.38]	2.76 [2.37, 3.02]
Sweden	6.52 [3.98, 15.83]	1.11 [0.42, 1.70]	2.59 [2.31, 2.87]
Switzerland	3.03 [2.00, 7.80]	1.02 [0.42, 1.48]	2.64 [2.34, 2.87]
United Kingdom	6.68 [4.21, 15.05]	0.97 [0.42, 1.39]	2.72 [2.44, 2.93]

Table S2: Model-estimated values for infection AR by July 4, 2020, IFRs, and reproductive numbers (R_0) for the investigated European countries. We report the median values with the 90% CI

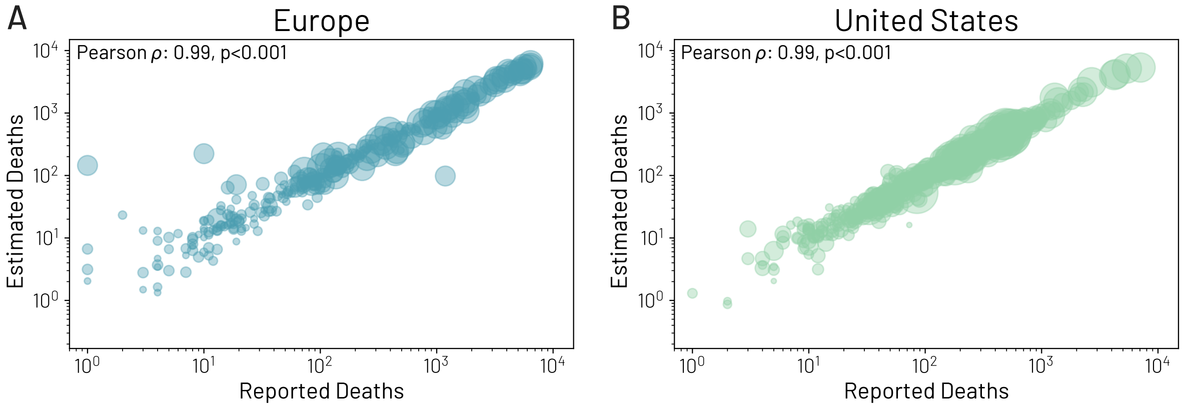

Figure S7: **Model calibration correlations.** The correlation between the median weekly estimated deaths fit using the wMAPE state/country level calibration method with a 25% tolerance threshold and the weekly reported deaths in Europe (A) and the US (B). Each circle represents a weekly value within the fitting window of the weeks between March 22, 2020 and June 27,2020 for a single country/state. For the US n = 504 and for Europe n = 280. The size of the circle is proportional to the population size of the country/state. The correlations are calculated using the Pearson correlation coefficient.

**Name**	Infection Attack Rate (%)	IFR (%)	R_0
Alabama	2.94 [1.68, 6.43]	1.01 [0.44, 1.53]	2.80 [2.51, 3.00]
Arizona	5.95 [3.04, 12.96]	0.95 [0.38, 1.59]	2.60 [2.38, 2.76]
Arkansas	1.52 [0.92, 4.07]	1.12 [0.39, 1.61]	2.64 [2.39, 2.93]
California	3.08 [1.40, 6.97]	0.83 [0.34, 1.40]	2.48 [2.24, 2.69]
Connecticut	9.44 [6.52, 19.60]	1.30 [0.63, 1.60]	2.80 [2.50, 3.04]
Delaware	8.09 [5.37, 20.86]	1.23 [0.48, 1.60]	2.78 [2.43, 3.11]
Florida	2.16 [1.29, 5.20]	1.17 [0.51, 1.80]	2.62 [2.36, 2.84]
Georgia	4.46 [2.64, 9.99]	0.89 [0.38, 1.29]	2.71 [2.45, 2.95]
Illinois	7.42 [4.41, 18.74]	1.02 [0.43, 1.47]	2.73 [2.41, 2.99]
Indiana	4.83 [2.94, 11.55]	1.07 [0.50, 1.49]	2.80 [2.52, 3.02]
Kansas	0.95 [0.57, 2.38]	0.95 [0.41, 1.42]	2.63 [2.37, 2.88]
Kentucky	1.68 [1.08, 4.16]	1.05 [0.43, 1.52]	2.79 [2.50, 2.97]
Louisiana	6.22 [4.26, 14.14]	1.13 [0.50, 1.46]	2.76 [2.40, 3.03]
Maryland	6.53 [3.83, 14.77]	1.03 [0.43, 1.48]	2.74 [2.46, 2.97]
Massachusetts	12.96 [7.80, 29.45]	1.15 [0.49, 1.59]	2.76 [2.52, 3.02]
Michigan	6.39 [4.11, 13.79]	1.03 [0.48, 1.46]	2.72 [2.43, 3.02]
Minnesota	5.76 [3.05, 13.29]	0.94 [0.35, 1.43]	2.76 [2.47, 2.94]
Mississippi	5.19 [3.23, 10.67]	1.16 [0.53, 1.54]	2.76 [2.54, 2.98]
Missouri	2.17 [1.38, 5.58]	1.03 [0.42, 1.46]	2.73 [2.45, 2.94]
Nebraska	2.89 [1.75, 7.12]	1.08 [0.44, 1.56]	2.71 [2.53, 2.99]
Nevada	2.74 [1.56, 6.68]	0.91 [0.40, 1.41]	2.62 [2.36, 2.86]
New Hampshire	6.63 [3.53, 15.18]	0.88 [0.39, 1.55]	2.55 [2.37, 2.77]
New Jersey	15.20 [10.22, 31.26]	1.20 [0.60, 1.55]	2.79 [2.49, 3.03]
New Mexico	3.55 [2.25, 8.78]	1.07 [0.45, 1.54]	2.76 [2.42, 2.97]
New York	13.37 [9.07, 26.72]	1.14 [0.56, 1.50]	2.78 [2.44, 3.02]
North Carolina	2.81 [1.53, 6.45]	0.97 [0.43, 1.50]	2.70 [2.42, 2.92]
Ohio	2.96 [1.67, 6.95]	1.12 [0.46, 1.58]	2.76 [2.52, 3.02]
Oklahoma	1.04 [0.71, 2.74]	1.04 [0.41, 1.44]	2.61 [2.32, 2.86]
Oregon	0.78 [0.48, 1.92]	1.08 [0.44, 1.56]	2.59 [2.37, 2.78]
Pennsylvania	5.56 [3.46, 12.26]	1.24 [0.57, 1.63]	2.80 [2.43, 3.04]
South Carolina	2.30 [1.36, 5.21]	0.98 [0.43, 1.51]	2.75 [2.49, 2.92]
Tennessee	1.42 [0.90, 3.46]	1.08 [0.42, 1.53]	2.60 [2.45, 2.89]
Texas	2.20 [1.20, 5.25]	0.81 [0.36, 1.27]	2.61 [2.33, 2.81]
Virginia	2.81 [1.56, 6.09]	0.96 [0.42, 1.46]	2.71 [2.42, 2.92]
Washington	1.84 [1.08, 4.28]	0.92 [0.41, 1.44]	2.64 [2.36, 2.88]
Wisconsin	1.71 [1.21, 4.46]	1.08 [0.41, 1.46]	2.66 [2.51, 2.97]

Table S3: Model-estimated values for the infection AR by July 4, 2020, IFR, and reproductive number (R_0) for the investigated US states. We report the median values with the 95% CI

text (i.e. a tolerance of 25%).

3 Sensitivity Analysis

3.1 Unconstrained pandemic evolution realizations. In the main text we report the calibration results where we constrain the simulation results to realizations where Italy is the first country, of those under investigation, to experience sustained, local transmission. This was in part due to the fact that in the early phase of the pandemic there was limited testing capacity, so confirmed deaths might be a better proxy for the relative start of local outbreaks in each country rather than reported cases. As of March 9, 2020, Italy reported 463 cumulative deaths, Spain 35, US 26, France 25, Germany 2, the UK 7, and Italy was the first in the region to impose a national lockdown. Here, we show the modeling results of the unconstrained realizations. In Fig. S8 we show the rank distributions illustrating the probability, in our simulations, that each country started the local outbreak in a particular order R (i.e., first, second, third etc.). While an initial start in the US and UK are the most likely scenarios in the ensemble (39% and 22% of simulations respectively), the empirical observations of case importation are also compatible with starts in other countries such as Germany, France, or Italy (13%, 10%, and 7% respectively). As a way to quantify and cluster the similarity of onset profiles, we compute and compare their cosine similarity. In particular, for each country, we create a vector where the x_R component is the fraction of runs in which that country started the local outbreak in R^{th} position. We then compute, for each pair, the cosine similarity building a similarity matrix. On the right side of Fig. S8 we show the correlation network with a threshold between pairs of 0.9. We use a community detection algorithm based on label propagation (37) that identifies three country clusters. These clusters represent country onset profiles that are considered to be similar to others in that group. The first group contains the US, UK, France, Germany, and Italy and these are all the countries among the first to have experienced the epidemic. The first confirmed cases in these countries were all reported within an eight day period. The second cluster instead is formed by countries such as Spain, Switzerland, Poland, and Portugal which are in the second group of countries to start observing local spreading of the virus. Spain acts as bridge with the first group. We find a third cluster, that includes all countries among the last to have experienced the epidemic such as Bulgaria, Iceland, and Lithuania. The first case detected within countries of this final cluster was on February 25, 2020 in Croatia (38), over a month after the first case was detected in Europe.

3.2 Alternative distance measure for model calibration. We further examine the robustness of the individual state and country results by testing an additional calibration method that still uses the $wMAPE$ distance measure in the ABC rejection algorithm with the same fitting window and priors on the IFR and reporting delays. However, rather than using an explicit tolerance threshold, like what is described in Section 2, we set the tolerance threshold to be a quantile of the empirical distance measure, $s(E', E)$, distribution (39). Specifically, we keep all realizations in the 0.05 quantile which leaves us with approximately 4,000 realizations. The results in this section, using this alternative distance measure, align with and confirm the results reported in the main text. In Fig. S9 we show the correlation of the weekly new deaths, infection ARs as of July 4, 2020, and estimated IFRs between the model calibrated using the $wMAPE$, ABC approach with a specific tolerance threshold of 25% (from main text) and this additional method where the tolerance threshold is the 0.05 quantile of the $wMAPE$ distance measure distribution.

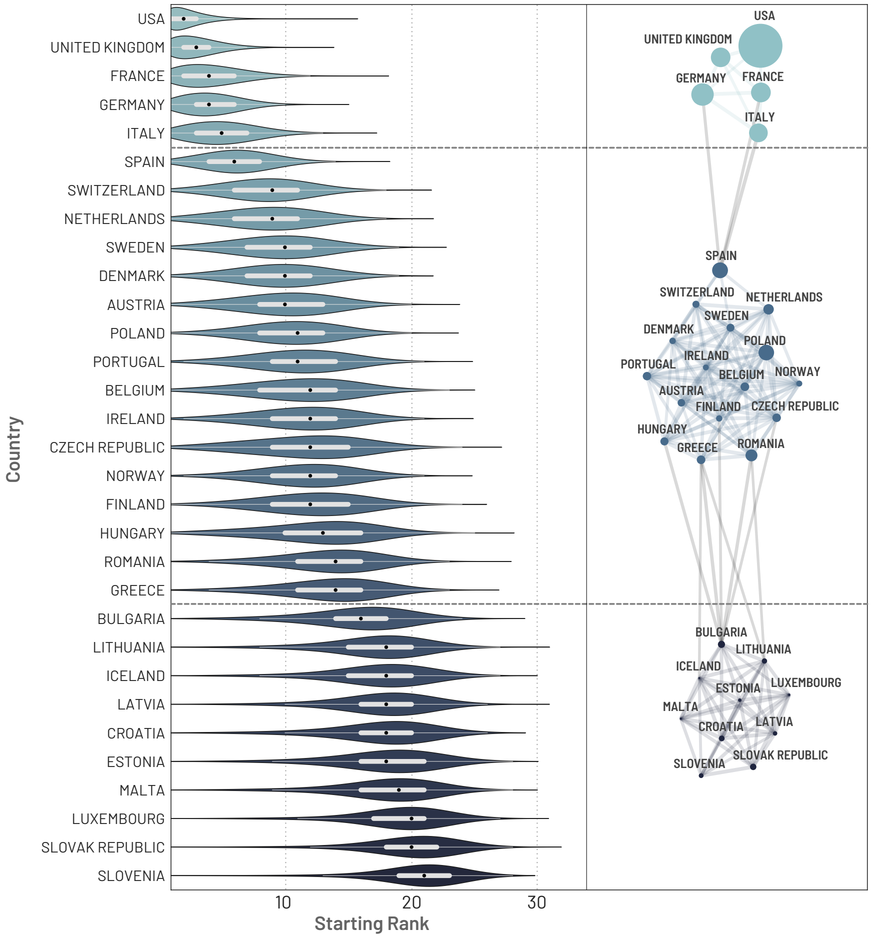

Figure S8: **Onset of local outbreaks in the selected ensemble.** On the left we show the distribution of starting ranks. The plot shows the probability that each country started the local outbreak in rank R respect to the others. Distribution are the result of the ABC analysis on 200,000 independent model realizations. The central dot corresponds to the median ad the lower and upper ends of the boxes are the 25% and 75% percentiles. The upper whisker is either the largest value but not larger than 1.5×IQR from the upper quartile and the lower whisker extends from the lower quartile to the smallest value at most 1.5×IQR. On the right we show the similarity network computed considering the cosine similarity of the starting rank distribution for each pair of countries. We threshold links showing just those equal or above to 0.9. The size of each node is proportional to the population of the respective country. The three clusters are identified via a community detection algorithm based on label propagation.

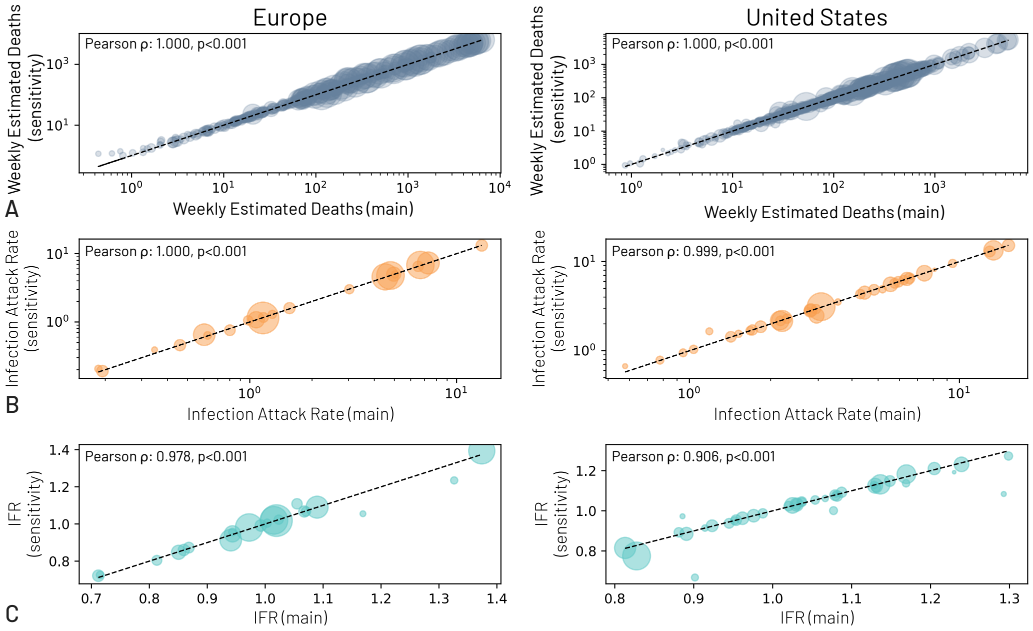

Figure S9: **Calibration comparison.** (A) The correlation between the weekly estimated deaths using the ABC calibration in the main text, with the 25% tolerance threshold (main) and the calibration described in section 3.2 which uses the quantile of the $wMAPE$ distance measure distribution (sensitivity). Each circle represents the weekly values for a single European country (left, n = 280) or US state (right, n = 504). (B) The correlation of the cumulative infection AR for each country/state as of July 4, 2020 between the two calibration methods for Europe (left, n = 20) and the US (right, n = 36) (C) The correlation of the estimated IFRs for each calibration method for Europe (left, n = 20) and US (right, n = 36). All circle sizes are proportional to the populations of each US state and European country. The dashed line in all figures represents the $y = x$ line. The correlations are calculated using the Pearson correlation coefficient ρ.

3.3 Overdispersion of disease transmission. Evidence has accrued that overdispersion in SARS-CoV-2 transmission is an important element of the COVID-19 pandemic. Overdispersion has been measured as a relevant feature in several specific studies (40; 41; 42; 43; 44; 45), that show that secondary infections are best characterized by a negative binomial distribution with an overdispersion parameter k, where lower values of k correspond to a broader distribution and differ from the classic Poisson distribution ($k = \infty$). Among those concerned with SARS-CoV-2 transmission dynamics, two papers estimate the overdispersion parameter from the analysis of primary infections and contact tracing data on a large scale. Sun et al. (41) reports the analysis of 1,178 SARS-CoV-2–infected individuals and their 15,648 close contacts, yielding an offspring distribution with an overdispersion parameter k=0.3 (95%CI [0.23 - 0.39]). A second study from Bi et al.(42) analyzes 391 SARS-CoV-2 cases and 1,286 close contacts, reporting an overdispersion parameter k=0.58 (95%CI [0.35 - 1.18]).

Our model is discrete, stochastic, spatial, and accounts for multiple sources of heterogeneity. The age structure of each subpopulation, contact patterns, setting and location, travel patterns by age and location, commuting patterns are examples. These heterogeneities lead to distributions of individual reproductive numbers that are overdispersed relative to the Poisson distribution that is generated when infectious individuals, settings, and populations are homogeneously distributed. Although the transmission dynamics will depend on the specific details of each population such as mobility constraints, NPIs, and days of the week, we empirically measured overdispersion in our model by looking at the offspring distribution of 1,000 introductions in the Wuhan area for one week in November, 2019 ($R_0 = 2.5$). In Fig. S10 A, we report the percentage of secondary infections produced by the corresponding percentage of primary infectors in our model and we compare them to negative binomial distributions with dispersion parameters $k \in \{0.3, 0.6, 2.0\}$, and a Poisson distribution (all having a mean of 2.5). It is readily observable that the model's disease transmission is far from a Poisson branching process, with about 25% of infections generating 70% of the transmission events. Although the results may vary for locations across the world, the 70-25 rule provides the overall characterization of GLEAM's overdispersion (for $R_0 = 2.5$).

However, the relevance of overdispersion at the global scale is much less clear where there may be hundreds of repeated introductions into a region over a short time period (40). Most results on super-spreading events are potentially altered by mitigation measures and observation biases (asymptomatic individuals, surveillance, etc.), and it is thus not guaranteed that these measurements would also hold when few or no mitigation measures are in place and/or in case of wider community spread. For this reason we provide a full sensitivity analysis of the model in which we fix the overdispersion for the transmission of primary infections to follow a negative binomial distribution with a dispersion parameter $k \in \{0.3, 0.6\}$ as from Ref.(41; 42; 46). This can be viewed as a proxy for other forms of heterogeneity not directly captured in the model structure, such as viral load. While using a specific overall dispersion in our model not differentiated across age groups, settings, locations, etc. is a strong assumption, it provides an indication of the stability of the results reported in this study.

To perform the sensitivity analysis we have implemented the GLEAM model by imposing an overdispersed transmission dynamic for the transition operators defined in Sec.1.1, yielding simulations with offspring distributions from primary cases best described by a negative binomial with a dispersion parameter $k = 0.3$. This model was then used to generate an ensemble of stochastic realizations of the global pandemic. The same ABC method described in Sec. 2 was used to calibrate the global, model results. We also performed an additional sensitivity analysis by repeating the entire analysis using an overdispersion parameter $k = 0.6$.

In Fig. S10 B) and C) we report the quantitative analyses of the results for the scenario with an overdispersion parameter $k = 0.3$. Specifically, we calculate the probability $p(t)$ of the time t of the onset of local transmission and calculate for each European country and US state the median time T and the standard deviation σ of the distribution in the model with overdispersion parameter $k = 0.3$ and compare them to the values obtained from the baseline GLEAM model (as reported in the main text). The estimated difference across European countries and US states for the median onset times, T, (at least 10 new, locally-generated infections per day) is about 3 days later in the new analysis. We also

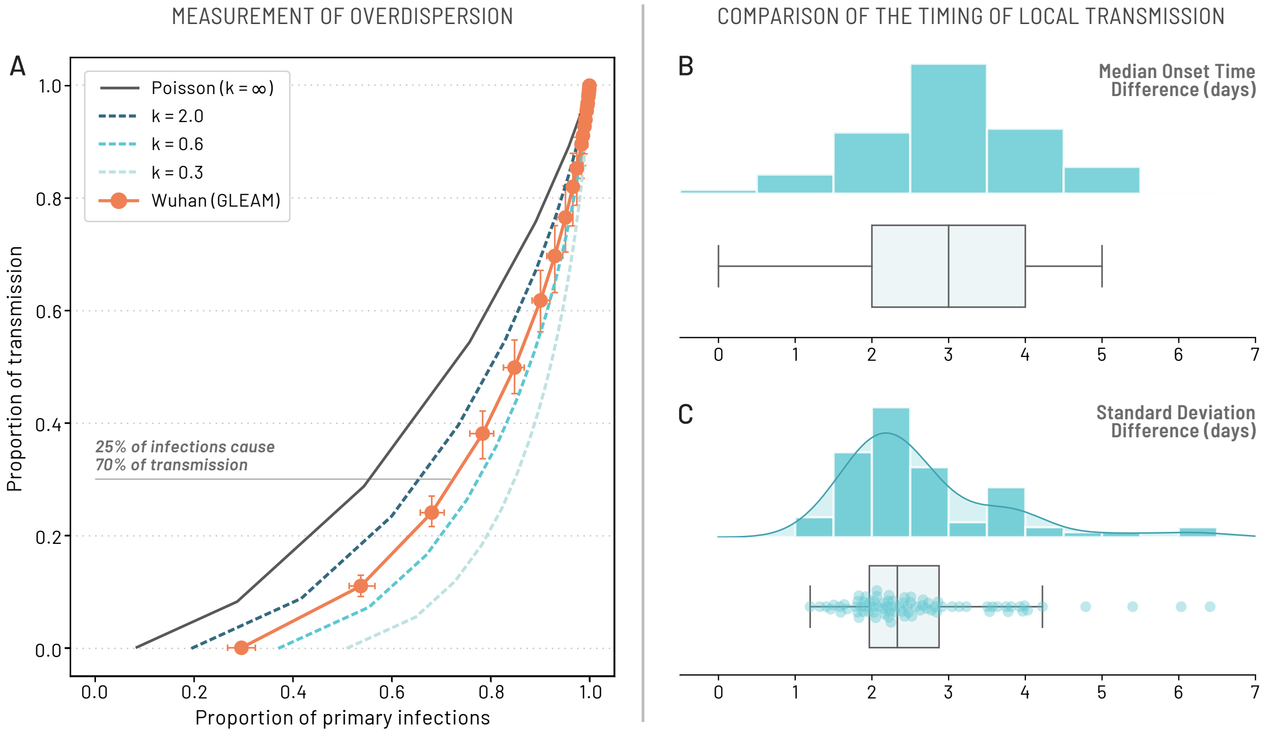

Figure S10: **Overdispersion of the disease transmission** (A) Empirical measurement of overdispersion in the model. We simulated for the geographical area of Wuhan (China), with an $R_0 = 2.5$, $1,000$ introductions and calculated the proportion of transmission attributable to a proportion of infections. The error bars represent the 90% CI of the uncertainty measured through a bootstrapping procedure that generates $n = 100$ sample distributions of 500 introductions over the full set of simulations. The GLEAM model shows that 25% of all primary infections are responsible for 70% of secondary infections within Wuhan. The other lines represent the proportion of transmission as a function of the proportion of infections in the case of exact negative binomial distributions with $k \in \{0.3, 0.6, 2.0\}$ (dotted lines) and the Poisson case ($k = \infty$) with mean 2.5 (solid line). B), C) Comparison of the distributions between the time of the onset of local transmission (10 locally-generated infections per day) of the GLEAM model (from the main text) and the model that uses, for all transmission events, a negative binomial offspring distribution with a dispersion parameter $k = 0.3$. We calculate for each European country and US state considered in the study the distribution of the timing of local transmission $p(t)$, and report the difference of the median time T (B) and standard deviation σ (C). Specifically, we subtract the GLEAM model in the main text from the model with an overdispersion parameter k=0.3 for all locations (each data point is a European country, n = 30 or US state n = 49. In (B) and (C) we show boxplots where the middle line is the median, the lower and upper ends of the boxes are the 25% and 75% percentiles respectively, the upper whisker is either the largest value but not larger than 1.5×IQR from the upper quartile and the lower whisker extends from the lower quartile to the smallest value at most 1.5×IQR.

measure a very modest increase in the standard deviation of the $p(t)$ distribution for the model with $k = 0.3$ of about 2.5 days for each country and state. These differences are practically not observable on the "epiweek" scale (7 days) over which real data are often averaged or reported. For the scenario with $k = 0.6$, the differences are smaller with a difference in the standard deviation of about 1 day.

These small, observed differences can be understood by considering that the time of the onset of local transmission is determined by the global circulation of SARS-CoV-2 that results in multiple, repeated introductions of infectious individuals in major locations at an increasing frequency in time due to the global growth of the epidemic. While overdispersion has been observed on single introductions, its effect is reduced when averaging over many transmission events of increasing frequency in time (40). In Fig. S11 we also show the full distribution of the time of onset of local transmission (10 locally-generated infections per day) for the scenario with $k = 0.3$ for all European countries and US states analyzed in the paper. The sensitivity analysis clearly indicates that the results are stable to values of additional overdispersion in the range of available empirically observed estimates.

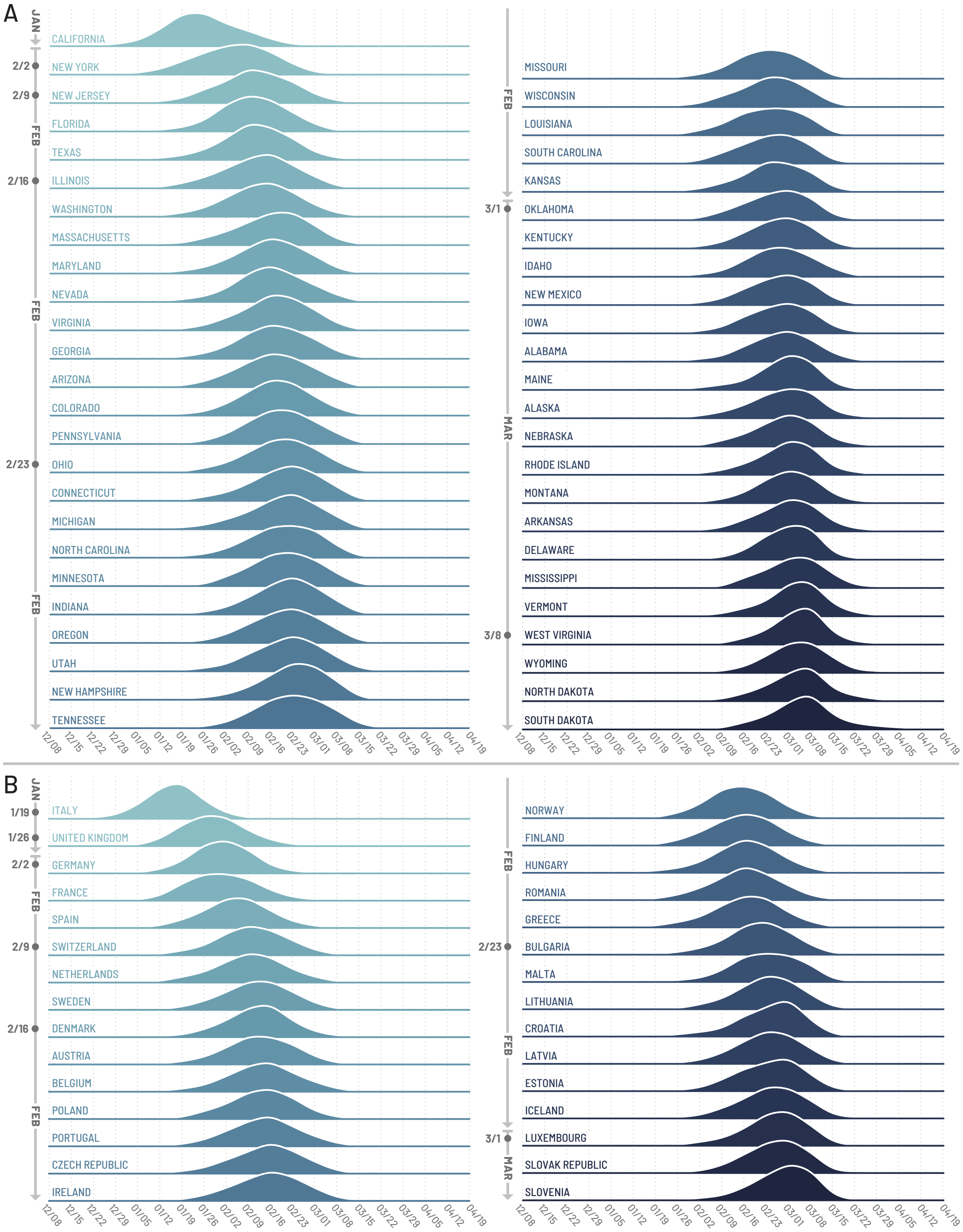

Figure S11: **Timing of the onset of local transmission in the model where all individual transmission events follow a negative binomial with dispersion parameter $\mathbf{k = 0.3}$.** Posterior distribution $p(t)$ of the week when each US state (A) or European country (B) first reached 10 locally generated SARS-CoV-2 transmission events per day.

4 SARS-CoV-2 Introduction Statistics

To generate Fig. 3 in the main text, we record all introduction events through April 30, 2020. We aggregate the observations from census areas to US states or European countries (i.e., the targets). Then we construct a directed and weighted network in which importation sources link the target states/countries. The width of the link is the average share of importations from each source across all runs selected through April 30, 2020. Using these values, we build the chord diagrams shown in the manuscript. Since the weight of each link is the average across all runs of the normalized share of importation per run, the sum of incoming links for each target is one. To help the readability of the plots, we aggregated sources considering macro areas such as Europe and Asia. We keep the US (to isolate the national importations) and mainland China (as the epicenter of the pandemic) separate. All the other sources are grouped together and labeled "Others". More specifically, source countries of importations in are grouped as:

- **Asia**: Afghanistan, Armenia, Azerbaijan, Bahrain, Bangladesh, Brunei, Cambodia, Cyprus, India, Indonesia, Iran, Iraq, Israel, Japan, Jordan, Kazakhstan, Korea, Kuwait, Kyrgyzstan, Lao PDR, Lebanon, Malaysia, Maldives, Mongolia, Myanmar, Nepal, Oman, Pakistan, Philippines, Qatar, Saudi Arabia, Singapore, Sri Lanka, Taiwan, Tajikistan, Thailand, Turkey, United Arab Emirates, Uzbekistan, Vietnam, Yemen

- **China**: mainland China

- **Europe**: Albania, Austria, Belarus, Belgium, Bosnia and Herzegovina, Bulgaria, Croatia, Czech Republic, Denmark, Estonia, Finland, France, Germany, Gibraltar, Greece, Hungary, Iceland, Ireland, Isle of Man, Italy, Jersey, Kosovo, Latvia, Lithuania, Luxembourg, North Macedonia, Malta, Moldova, Montenegro, Netherlands, Norway, Poland, Portugal, Romania, Russian Federation, Serbia, Slovak Republic, Slovenia, Spain, Sweden, Switzerland, Ukraine, United Kingdom

- **Others**: Algeria, Angola, Antigua and Barbuda, Argentina, Aruba, Australia, Bahamas, Barbados, Belize, Bermuda, Bolivia, Botswana, Brazil, British Virgin Islands, Burundi, Cameroon, Canada, Cape Verde, Caribbean Netherlands, Cayman Islands, Chile, Colombia, Congo, Cook Islands, Costa Rica, Cuba, Curaçao, Côte d'Ivoire, Djibouti, Dominica, Dominican Republic, Ecuador, Egypt, El Salvador, Equatorial Guinea, Ethiopia, Fiji, French Guiana, French Polynesia, Gambia, Ghana, Greenland, Grenada, Guadeloupe, Guatemala, Guinea, Guyana, Haiti, Honduras, Jamaica, Kenya, Liberia, Madagascar, Martinique, Mauritius, Mexico, Morocco, Mozambique, Namibia, New Zealand, Nicaragua, Nigeria, Palau, Panama, Paraguay, Peru, Rwanda, Samoa, Senegal, Seychelles, Sierra Leone, Somalia, South Africa, South Sudan, St-Barthélemy, St. Kitts and Nevis, St. Lucia, St. Maarten, St. Vincent and Grenadines, Sudan, Suriname, Tonga, Trinidad and Tobago, Tunisia, Turks and Caicos Islands, Uganda, Uruguay, Vanuatu, Venezuela, Zambia, Zanzibar, Zimbabwe

- **United States**: all the US states plus the US territories (American Samoa, Guam, Northern Mariana Islands, Puerto Rico, U.S. Virgin Islands)

In Table S4 we report the share of introduction of SARS-CoV-2 infections for all European countries that experienced a local outbreak considering all the infections imported up to April 30, 2020. Compared with the seeding events networks (see section 5), the flows are radically different, especially for the early states and countries experiencing local transmission. The critical role of China before the travel restrictions of January 23, is replaced by a much larger fraction of introduction events of domestic or nearby countries origin.

In Table S5 we report the share of introduction of SARS-Cov-2 infections for all US states considering all the infections imported up to April 30, 2020.

State	Europe	China	Asia	USA	Others
Italy	0.69 (0.6, 0.8)	<0.01 (<0.01, <0.01)	0.21 (0.13, 0.28)	0.06 (0.02, 0.06)	0.04 (0.02, 0.05)
United Kingdom	0.58 (0.48, 0.68)	<0.01 (<0.01, <0.01)	0.27 (0.19, 0.35)	0.08 (0.03, 0.09)	0.07 (0.03, 0.08)
Germany	0.53 (0.42, 0.64)	<0.01 (<0.01, <0.01)	0.38 (0.27, 0.48)	0.04 (0.02, 0.05)	0.04 (0.02, 0.05)
France	0.53 (0.42, 0.63)	<0.01 (<0.01, <0.01)	0.27 (0.18, 0.35)	0.06 (0.02, 0.07)	0.15 (0.1, 0.18)
Spain	0.84 (0.79, 0.91)	<0.01 (0.0, <0.01)	0.10 (0.05, 0.12)	0.04 (0.01, 0.04)	0.02 (<0.01, 0.03)
Switzerland	0.54 (0.42, 0.64)	<0.01 (0.0, <0.01)	0.38 (0.27, 0.48)	0.05 (0.02, 0.06)	0.04 (0.01, 0.04)
Netherlands	0.58 (0.47, 0.69)	<0.01 (0.0, <0.01)	0.25 (0.16, 0.32)	0.06 (0.02, 0.07)	0.12 (0.06, 0.15)
Sweden	0.67 (0.56, 0.77)	<0.01 (0.0, <0.01)	0.28 (0.18, 0.37)	0.04 (0.01, 0.04)	0.02 (<0.01, 0.02)
Denmark	0.54 (0.42, 0.65)	<0.01 (0.0, 0.0)	0.39 (0.27, 0.5)	0.05 (0.02, 0.05)	0.03 (<0.01, 0.03)
Austria	0.53 (0.42, 0.64)	<0.01 (0.0, <0.01)	0.41 (0.3, 0.51)	0.03 (0.01, 0.04)	0.03 (0.01, 0.04)
Belgium	0.57 (0.46, 0.69)	<0.01 (0.0, 0.0)	0.33 (0.22, 0.43)	0.05 (0.02, 0.06)	0.04 (0.02, 0.05)
Poland	0.73 (0.65, 0.83)	<0.01 (0.0, 0.0)	0.21 (0.13, 0.28)	0.03 (<0.01, 0.04)	0.02 (<0.01, 0.03)
Portugal	0.84 (0.79, 0.91)	<0.01 (0.0, 0.0)	0.07 (0.04, 0.1)	0.04 (0.01, 0.04)	0.05 (0.01, 0.05)
Czech Republic	0.62 (0.52, 0.72)	<0.01 (0.0, 0.0)	0.31 (0.21, 0.4)	0.04 (0.01, 0.04)	0.04 (0.01, 0.04)
Ireland	0.75 (0.67, 0.85)	<0.01 (0.0, 0.0)	0.11 (0.06, 0.14)	0.10 (0.04, 0.12)	0.05 (0.01, 0.05)
Norway	0.75 (0.67, 0.84)	<0.01 (0.0, 0.0)	0.22 (0.13, 0.29)	0.03 (<0.01, 0.03)	0.01 (<0.01, 0.01)
Finland	0.63 (0.53, 0.74)	<0.01 (0.0, 0.0)	0.32 (0.22, 0.42)	0.03 (<0.01, 0.03)	0.02 (<0.01, 0.02)
Hungary	0.70 (0.62, 0.81)	<0.01 (0.0, <0.01)	0.23 (0.14, 0.29)	0.04 (0.01, 0.05)	0.03 (<0.01, 0.03)
Romania	0.76 (0.69, 0.85)	<0.01 (0.0, 0.0)	0.20 (0.11, 0.25)	0.02 (<0.01, 0.03)	0.02 (<0.01, 0.02)
Greece	0.79 (0.73, 0.87)	<0.01 (0.0, 0.0)	0.14 (0.08, 0.17)	0.05 (0.01, 0.05)	0.03 (<0.01, 0.03)
Bulgaria	0.72 (0.65, 0.82)	<0.01 (0.0, 0.0)	0.23 (0.14, 0.3)	0.03 (<0.01, 0.03)	0.02 (<0.01, 0.02)
Malta	0.90 (0.87, 0.95)	<0.01 (0.0, 0.0)	0.07 (0.03, 0.09)	0.01 (<0.01, 0.01)	0.02 (<0.01, 0.02)
Lithuania	0.81 (0.75, 0.89)	<0.01 (0.0, 0.0)	0.17 (0.1, 0.22)	0.02 (0.0, 0.02)	<0.01 (0.0, <0.01)
Croatia	0.73 (0.65, 0.82)	<0.01 (0.0, 0.0)	0.18 (0.11, 0.23)	0.04 (0.01, 0.05)	0.05 (0.01, 0.06)
Latvia	0.74 (0.67, 0.83)	<0.01 (0.0, 0.0)	0.23 (0.14, 0.29)	0.02 (0.0, 0.03)	0.01 (0.0, 0.02)
Estonia	0.65 (0.55, 0.76)	<0.01 (0.0, 0.0)	0.31 (0.21, 0.41)	0.03 (<0.01, 0.03)	<0.01 (0.0, 0.01)
Iceland	0.69 (0.6, 0.82)	<0.01 (0.0, 0.0)	0.07 (0.03, 0.09)	0.16 (0.07, 0.2)	0.08 (0.02, 0.1)
Luxembourg	0.82 (0.76, 0.89)	<0.01 (0.0, 0.0)	0.13 (0.07, 0.17)	0.03 (<0.01, 0.04)	0.02 (<0.01, 0.02)
Slovak Republic	0.90 (0.86, 0.96)	<0.01 (0.0, 0.0)	0.09 (0.03, 0.12)	<0.01 (0.0, 0.0)	<0.01 (0.0, <0.01)
Slovenia	0.71 (0.62, 0.82)	<0.01 (0.0, 0.0)	0.24 (0.14, 0.31)	0.04 (0.0, 0.04)	0.02 (0.0, 0.02)

Table S4: Introduction of SARS-CoV-2 infections through April 30. Sources are listed from the second column on. Targets are the European countries listed in the first column. Numbers are rounded to the second digit.

State	USA	China	Asia	Europe	Others
California	0.69 (0.6, 0.81)	<0.01 (<0.01, <0.01)	0.11 (0.05, 0.15)	0.05 (0.02, 0.07)	0.14 (0.07, 0.19)
New York	0.56 (0.43, 0.67)	<0.01 (<0.01, <0.01)	0.11 (0.05, 0.15)	0.14 (0.07, 0.19)	0.19 (0.12, 0.23)
New Jersey	0.56 (0.44, 0.68)	<0.01 (<0.01, <0.01)	0.10 (0.05, 0.14)	0.13 (0.06, 0.18)	0.20 (0.12, 0.24)
Florida	0.71 (0.62, 0.82)	<0.01 (0.0, <0.01)	0.03 (0.01, 0.03)	0.07 (0.03, 0.1)	0.20 (0.11, 0.24)
Texas	0.78 (0.71, 0.87)	<0.01 (0.0, <0.01)	0.05 (0.02, 0.07)	0.04 (0.02, 0.05)	0.12 (0.06, 0.15)
Illinois	0.71 (0.63, 0.82)	<0.01 (0.0, <0.01)	0.07 (0.03, 0.1)	0.06 (0.03, 0.08)	0.15 (0.08, 0.18)
Washington	0.81 (0.74, 0.89)	<0.01 (0.0, <0.01)	0.06 (0.02, 0.08)	0.03 (0.01, 0.04)	0.10 (0.04, 0.12)
Massachusetts	0.69 (0.6, 0.8)	<0.01 (0.0, <0.01)	0.05 (0.02, 0.07)	0.10 (0.04, 0.14)	0.15 (0.09, 0.19)
Maryland	0.71 (0.62, 0.82)	<0.01 (0.0, <0.01)	0.08 (0.04, 0.11)	0.08 (0.03, 0.1)	0.12 (0.07, 0.15)
Nevada	0.78 (0.71, 0.89)	<0.01 (0.0, 0.0)	0.03 (<0.01, 0.03)	0.04 (0.02, 0.05)	0.15 (0.05, 0.2)
Virginia	0.75 (0.67, 0.84)	<0.01 (0.0, <0.01)	0.07 (0.03, 0.09)	0.06 (0.03, 0.08)	0.12 (0.07, 0.15)
Georgia	0.78 (0.7, 0.86)	<0.01 (0.0, 0.0)	0.07 (0.03, 0.09)	0.05 (0.02, 0.06)	0.10 (0.06, 0.13)
Arizona	0.82 (0.77, 0.92)	<0.01 (0.0, <0.01)	0.02 (<0.01, 0.03)	0.02 (<0.01, 0.03)	0.13 (0.05, 0.17)
Colorado	0.83 (0.77, 0.9)	<0.01 (0.0, 0.0)	0.02 (<0.01, 0.03)	0.04 (0.01, 0.05)	0.12 (0.06, 0.15)
Pennsylvania	0.78 (0.72, 0.86)	<0.01 (0.0, 0.0)	0.02 (<0.01, 0.03)	0.05 (0.02, 0.07)	0.14 (0.08, 0.18)
Ohio	0.81 (0.76, 0.89)	<0.01 (0.0, <0.01)	0.03 (<0.01, 0.03)	0.04 (0.01, 0.05)	0.12 (0.07, 0.15)
Connecticut	0.64 (0.54, 0.76)	<0.01 (<0.01, <0.01)	0.07 (0.03, 0.1)	0.10 (0.04, 0.14)	0.18 (0.11, 0.22)
Michigan	0.79 (0.73, 0.87)	<0.01 (0.0, 0.0)	0.03 (0.01, 0.04)	0.05 (0.02, 0.06)	0.13 (0.07, 0.16)
North Carolina	0.81 (0.75, 0.89)	<0.01 (0.0, 0.0)	0.03 (<0.01, 0.03)	0.05 (0.02, 0.06)	0.11 (0.06, 0.14)
Minnesota	0.77 (0.69, 0.85)	<0.01 (0.0, 0.0)	0.04 (0.02, 0.06)	0.04 (0.01, 0.05)	0.16 (0.09, 0.19)
Indiana	0.78 (0.71, 0.87)	<0.01 (0.0, <0.01)	0.04 (0.02, 0.05)	0.05 (0.02, 0.06)	0.13 (0.07, 0.16)
Oregon	0.84 (0.79, 0.92)	<0.01 (0.0, 0.0)	0.04 (0.01, 0.05)	0.02 (<0.01, 0.03)	0.10 (0.04, 0.12)
Utah	0.86 (0.82, 0.93)	<0.01 (0.0, 0.0)	0.03 (<0.01, 0.04)	0.03 (<0.01, 0.04)	0.08 (0.04, 0.11)
New Hampshire	0.68 (0.58, 0.79)	<0.01 (0.0, <0.01)	0.05 (0.02, 0.07)	0.11 (0.05, 0.14)	0.15 (0.09, 0.19)
Tennessee	0.82 (0.76, 0.9)	<0.01 (0.0, 0.0)	0.02 (<0.01, 0.03)	0.04 (0.01, 0.05)	0.12 (0.06, 0.15)
Missouri	0.82 (0.76, 0.89)	<0.01 (0.0, 0.0)	0.02 (<0.01, 0.03)	0.03 (<0.01, 0.04)	0.13 (0.07, 0.17)
Wisconsin	0.85 (0.8, 0.92)	<0.01 (0.0, 0.0)	<0.01 (<0.01, 0.01)	0.02 (<0.01, 0.03)	0.12 (0.06, 0.16)
Louisiana	0.84 (0.79, 0.91)	<0.01 (0.0, 0.0)	0.02 (<0.01, 0.03)	0.04 (0.01, 0.05)	0.11 (0.05, 0.13)
South Carolina	0.83 (0.78, 0.91)	<0.01 (0.0, 0.0)	0.02 (<0.01, 0.03)	0.04 (0.01, 0.06)	0.10 (0.05, 0.13)
Kansas	0.84 (0.79, 0.91)	<0.01 (0.0, 0.0)	0.02 (<0.01, 0.02)	0.03 (<0.01, 0.04)	0.12 (0.06, 0.14)
Oklahoma	0.83 (0.78, 0.91)	<0.01 (0.0, 0.0)	0.03 (<0.01, 0.03)	0.03 (<0.01, 0.04)	0.11 (0.06, 0.14)
Kentucky	0.82 (0.76, 0.9)	<0.01 (0.0, 0.0)	0.03 (<0.01, 0.04)	0.04 (0.01, 0.05)	0.12 (0.06, 0.15)
Idaho	0.88 (0.84, 0.94)	<0.01 (0.0, 0.0)	0.02 (<0.01, 0.02)	0.02 (<0.01, 0.02)	0.08 (0.03, 0.11)
New Mexico	0.89 (0.86, 0.95)	<0.01 (0.0, 0.0)	0.01 (<0.01, 0.02)	0.02 (<0.01, 0.03)	0.07 (0.03, 0.09)
Iowa	0.82 (0.76, 0.89)	<0.01 (0.0, 0.0)	0.02 (<0.01, 0.03)	0.03 (<0.01, 0.04)	0.13 (0.07, 0.17)
Alabama	0.70 (0.61, 0.83)	<0.01 (0.0, 0.0)	0.18 (0.08, 0.24)	0.03 (<0.01, 0.04)	0.09 (0.04, 0.11)
Maine	0.81 (0.75, 0.89)	<0.01 (0.0, <0.01)	0.02 (<0.01, 0.03)	0.04 (0.02, 0.06)	0.12 (0.06, 0.16)
Alaska	0.85 (0.8, 0.94)	<0.01 (0.0, 0.0)	0.04 (<0.01, 0.05)	0.02 (0.0, 0.02)	0.09 (0.03, 0.12)
Nebraska	0.82 (0.76, 0.9)	<0.01 (0.0, 0.0)	0.02 (<0.01, 0.03)	0.03 (<0.01, 0.04)	0.13 (0.07, 0.17)
Rhode Island	0.87 (0.83, 0.94)	<0.01 (0.0, 0.0)	<0.01 (0.0, 0.0)	0.05 (<0.01, 0.06)	0.08 (0.04, 0.11)
Montana	0.89 (0.85, 0.95)	<0.01 (0.0, 0.0)	<0.01 (<0.01, 0.01)	0.02 (<0.01, 0.02)	0.09 (0.04, 0.11)
Arkansas	0.84 (0.79, 0.91)	<0.01 (0.0, 0.0)	0.03 (<0.01, 0.03)	0.03 (<0.01, 0.04)	0.10 (0.05, 0.13)
Delaware	0.75 (0.67, 0.84)	<0.01 (0.0, 0.0)	0.02 (<0.01, 0.03)	0.06 (0.02, 0.08)	0.17 (0.1, 0.21)
Mississippi	0.85 (0.8, 0.92)	<0.01 (0.0, 0.0)	0.01 (<0.01, 0.02)	0.04 (<0.01, 0.05)	0.10 (0.04, 0.13)
Vermont	0.87 (0.83, 0.94)	<0.01 (0.0, 0.0)	0.02 (0.0, 0.02)	0.02 (0.0, 0.02)	0.09 (0.04, 0.12)
West Virginia	0.86 (0.81, 0.93)	<0.01 (0.0, 0.0)	0.01 (<0.01, 0.01)	0.03 (<0.01, 0.04)	0.10 (0.04, 0.13)
Wyoming	0.86 (0.81, 0.95)	<0.01 (0.0, 0.0)	0.05 (<0.01, 0.06)	0.02 (<0.01, 0.02)	0.07 (0.02, 0.09)
North Dakota	0.88 (0.85, 0.94)	<0.01 (0.0, 0.0)	<0.01 (0.0, 0.0)	<0.01 (0.0, <0.01)	0.11 (0.05, 0.14)
South Dakota	0.85 (0.8, 0.93)	<0.01 (0.0, 0.0)	<0.01 (0.0, 0.0)	0.02 (0.0, 0.02)	0.13 (0.06, 0.17)

Table S5: Introduction of SARS-Cov-2 infections through April 30. Sources are listed from the second column on. Targets are the US states listed in the first column. Numbers are rounded to the second digit.

Country	Europe	China	Asia	USA	Others
Italy	0.10 (0.0, 0.2)	0.72 (0.5, 1.0)	0.16 (0.0, 0.29)	<0.01 (0.0, 0.0)	<0.01 (0.0, 0.0)
United Kingdom	0.15 (0.0, 0.23)	0.52 (0.31, 0.71)	0.28 (0.1, 0.43)	0.02 (0.0, 0.0)	0.04 (0.0, 0.0)
Germany	0.22 (0.0, 0.33)	0.31 (0.11, 0.5)	0.43 (0.23, 0.62)	0.02 (0.0, 0.0)	0.02 (0.0, 0.0)
France	0.22 (0.0, 0.33)	0.41 (0.17, 0.62)	0.33 (0.11, 0.5)	0.02 (0.0, 0.0)	0.02 (0.0, 0.0)
Spain	0.55 (0.38, 0.75)	0.15 (0.0, 0.2)	0.26 (0.08, 0.4)	0.02 (0.0, 0.0)	0.02 (0.0, 0.0)
Switzerland	0.32 (0.12, 0.5)	0.09 (0.0, 0.11)	0.52 (0.33, 0.73)	0.03 (0.0, 0.0)	0.03 (0.0, 0.0)
Netherlands	0.39 (0.2, 0.57)	0.12 (0.0, 0.14)	0.42 (0.22, 0.6)	0.03 (0.0, 0.0)	0.04 (0.0, 0.0)
Sweden	0.32 (0.15, 0.5)	0.07 (0.0, 0.05)	0.56 (0.38, 0.75)	0.03 (0.0, 0.0)	0.02 (0.0, 0.0)
Denmark	0.33 (0.14, 0.5)	0.06 (0.0, 0.0)	0.57 (0.4, 0.76)	0.03 (0.0, 0.0)	0.02 (0.0, 0.0)
Austria	0.34 (0.14, 0.5)	0.07 (0.0, 0.04)	0.55 (0.36, 0.75)	0.02 (0.0, 0.0)	0.02 (0.0, 0.0)
Belgium	0.47 (0.25, 0.67)	0.07 (0.0, 0.0)	0.41 (0.2, 0.6)	0.03 (0.0, 0.0)	0.02 (0.0, 0.0)
Poland	0.55 (0.35, 0.75)	0.03 (0.0, 0.0)	0.39 (0.18, 0.56)	0.02 (0.0, 0.0)	0.02 (0.0, 0.0)
Portugal	0.70 (0.57, 0.89)	0.05 (0.0, 0.0)	0.19 (0.0, 0.3)	0.02 (0.0, 0.0)	0.04 (0.0, 0.0)
Czech Republic	0.40 (0.18, 0.6)	0.09 (0.0, 0.0)	0.47 (0.25, 0.68)	0.02 (0.0, 0.0)	0.02 (0.0, 0.0)
Ireland	0.56 (0.38, 0.76)	0.05 (0.0, 0.0)	0.25 (0.06, 0.38)	0.07 (0.0, 0.1)	0.07 (0.0, 0.09)
Norway	0.47 (0.3, 0.64)	0.01 (0.0, 0.0)	0.48 (0.32, 0.67)	0.03 (0.0, 0.0)	0.02 (0.0, 0.0)
Finland	0.37 (0.2, 0.53)	0.03 (0.0, 0.0)	0.56 (0.38, 0.75)	0.02 (0.0, 0.0)	0.02 (0.0, 0.0)
Hungary	0.50 (0.29, 0.71)	0.09 (0.0, 0.02)	0.38 (0.17, 0.57)	0.02 (0.0, 0.0)	0.02 (0.0, 0.0)
Romania	0.65 (0.5, 0.86)	0.02 (0.0, 0.0)	0.30 (0.11, 0.45)	0.01 (0.0, 0.0)	0.01 (0.0, 0.0)
Greece	0.60 (0.43, 0.8)	0.02 (0.0, 0.0)	0.32 (0.12, 0.5)	0.03 (0.0, 0.0)	0.02 (0.0, 0.0)
Bulgaria	0.60 (0.43, 0.8)	0.01 (0.0, 0.0)	0.36 (0.17, 0.5)	0.02 (0.0, 0.0)	0.01 (0.0, 0.0)
Malta	0.79 (0.67, 1.0)	<0.01 (0.0, 0.0)	0.18 (0.0, 0.25)	0.01 (0.0, 0.0)	0.02 (0.0, 0.0)
Lithuania	0.69 (0.56, 0.87)	<0.01 (0.0, 0.0)	0.29 (0.11, 0.42)	0.01 (0.0, 0.0)	<0.01 (0.0, 0.0)
Croatia	0.46 (0.25, 0.68)	<0.01 (0.0, 0.0)	0.45 (0.21, 0.67)	0.03 (0.0, 0.0)	0.05 (0.0, 0.04)
Latvia	0.57 (0.4, 0.76)	<0.01 (0.0, 0.0)	0.39 (0.2, 0.56)	0.02 (0.0, 0.0)	0.01 (0.0, 0.0)
Estonia	0.42 (0.21, 0.6)	<0.01 (0.0, 0.0)	0.55 (0.36, 0.75)	0.02 (0.0, 0.0)	<0.01 (0.0, 0.0)
Iceland	0.61 (0.46, 0.8)	0.01 (0.0, 0.0)	0.16 (0.0, 0.23)	0.14 (0.0, 0.2)	0.08 (0.0, 0.1)
Luxembourg	0.71 (0.58, 0.89)	<0.01 (0.0, 0.0)	0.24 (0.08, 0.33)	0.03 (0.0, 0.0)	0.02 (0.0, 0.0)
Slovak Republic	0.85 (0.79, 1.0)	<0.01 (0.0, 0.0)	0.14 (0.0, 0.2)	<0.01 (0.0, 0.0)	<0.01 (0.0, 0.0)
Slovenia	0.56 (0.4, 0.75)	<0.01 (0.0, 0.0)	0.38 (0.18, 0.56)	0.03 (0.0, 0.0)	0.02 (0.0, 0.0)

Table S6: Importation of seeding events. Sources are listed from the second column on. Targets are the countries listed in the first column. Numbers are rounded to the second digit.

5 SARS-CoV-2 Seeding Networks

In the main text we also discuss the differences between the full volume of SARS-CoV-2 introductions and the introduction events that could be relevant to the early onset of local transmission in each stochastic realization of the model. We generate a similar set of "introduction networks", we will refer to as seeding networks, to investigate the importance of different introduction sources before local transmission was established.

We generate the seeding networks as follows. As a first step, we track potential seeding events by air transportation (considering both individuals in the latent and infectious compartments) in any census areas of the US and Europe in all the runs selected. We then compute the day, in each run, in which the number of daily transitions from S to L is at least 10 in each state. In other words, we evaluate the date, in each run, when the state experienced the first local outbreak. We then track, in each run, the arrivals of latent and infectious individuals before or at the time of the local outbreak. From this standpoint, we build the seeding networks aggregating sources as described above.

In Table S6 we report the seeding share for all European countries considered (see also Fig. S12-B). Interestingly, China is the dominant seeding source for the first countries to have experienced the local outbreak such as Italy, UK, Germany, France and Spain. As we move down the list, towards countries that experienced a later start of the local outbreak, the share of seeding events from China rapidly decreases. Asia is a key source of infections for most of countries. For Denmark (57% [IQR 40% – 76%]), Finland (56% [IQR 38% – 75%]), Sweden (56% [IQR 38% – 75%]), Austria (55% [IQR 36% – 75%]), Estonia (55% [IQR 36% – 75%]) and Switzerland (52% [IQR 33% – 73%]) the share of importations from Asian countries is above 50%. However, the role of European seeding sources becomes more evident as we move

State	USA	China	Asia	Europe	Others
California	0.09 (0.0, 0.14)	0.74 (0.6, 1.0)	0.13 (0.0, 0.2)	0.01 (0.0, 0.0)	0.03 (0.0, 0.0)
New York	0.20 (0.0, 0.32)	0.45 (0.15, 0.71)	0.21 (0.0, 0.33)	0.09 (0.0, 0.14)	0.05 (0.0, 0.06)
New Jersey	0.28 (0.11, 0.42)	0.28 (0.07, 0.43)	0.25 (0.1, 0.37)	0.12 (0.0, 0.17)	0.07 (0.0, 0.1)
Florida	0.59 (0.42, 0.8)	0.05 (0.0, 0.0)	0.13 (0.0, 0.2)	0.11 (0.0, 0.17)	0.12 (0.0, 0.17)
Texas	0.57 (0.39, 0.79)	0.11 (0.0, 0.11)	0.21 (0.0, 0.33)	0.04 (0.0, 0.06)	0.07 (0.0, 0.1)
Illinois	0.46 (0.24, 0.68)	0.19 (0.0, 0.23)	0.22 (0.0, 0.34)	0.06 (0.0, 0.08)	0.07 (0.0, 0.1)
Washington	0.56 (0.38, 0.78)	0.11 (0.0, 0.11)	0.23 (0.04, 0.36)	0.03 (0.0, 0.03)	0.07 (0.0, 0.08)
Massachusetts	0.49 (0.29, 0.71)	0.16 (0.0, 0.17)	0.17 (0.0, 0.26)	0.11 (0.0, 0.17)	0.08 (0.0, 0.11)
Maryland	0.48 (0.28, 0.7)	0.11 (0.0, 0.09)	0.25 (0.07, 0.37)	0.09 (0.0, 0.14)	0.07 (0.0, 0.09)
Nevada	0.63 (0.47, 0.83)	0.04 (0.0, 0.0)	0.17 (0.0, 0.25)	0.04 (0.0, 0.06)	0.12 (0.0, 0.17)
Virginia	0.55 (0.37, 0.75)	0.08 (0.0, 0.06)	0.23 (0.07, 0.33)	0.08 (0.0, 0.12)	0.07 (0.0, 0.08)
Georgia	0.62 (0.46, 0.83)	0.07 (0.0, 0.0)	0.19 (<0.01, 0.29)	0.06 (0.0, 0.08)	0.06 (0.0, 0.08)
Arizona	0.71 (0.57, 0.92)	0.03 (0.0, <0.01)	0.11 (<0.01, 0.15)	0.03 (0.0, <0.01)	0.12 (0.0, 0.17)
Colorado	0.75 (0.64, 0.92)	0.01 (0.0, 0.0)	0.10 (0.0, 0.14)	0.04 (0.0, 0.06)	0.09 (0.0, 0.12)
Pennsylvania	0.71 (0.56, 0.9)	0.02 (0.0, 0.0)	0.10 (0.0, 0.15)	0.08 (0.0, 0.12)	0.09 (0.0, 0.12)
Ohio	0.71 (0.58, 0.9)	0.03 (0.0, <0.01)	0.13 (<0.01, 0.19)	0.06 (0.0, 0.08)	0.07 (0.0, 0.1)
Connecticut	0.45 (0.24, 0.65)	0.08 (0.01, 0.08)	0.23 (0.09, 0.33)	0.14 (0.04, 0.19)	0.10 (0.01, 0.12)
Michigan	0.63 (0.49, 0.85)	0.08 (0.0, 0.0)	0.16 (0.0, 0.23)	0.07 (0.0, 0.1)	0.07 (0.0, 0.1)
North Carolina	0.68 (0.54, 0.88)	0.06 (0.0, 0.0)	0.13 (0.0, 0.18)	0.07 (0.0, 0.09)	0.07 (0.0, 0.09)
Minnesota	0.68 (0.51, 0.87)	0.01 (0.0, 0.0)	0.16 (0.0, 0.25)	0.05 (0.0, 0.07)	0.10 (0.0, 0.13)
Indiana	0.66 (0.5, 0.85)	0.04 (0.0, 0.02)	0.16 (0.04, 0.22)	0.06 (0.0, 0.08)	0.08 (0.0, 0.1)
Oregon	0.73 (0.61, 0.9)	0.03 (0.0, 0.0)	0.15 (<0.01, 0.22)	0.02 (0.0, 0.02)	0.07 (0.0, 0.09)
Utah	0.79 (0.67, 0.96)	<0.01 (0.0, 0.0)	0.10 (0.0, 0.14)	0.04 (0.0, 0.05)	0.07 (0.0, 0.09)
New Hampshire	0.53 (0.36, 0.72)	0.09 (0.0, 0.06)	0.16 (0.04, 0.23)	0.13 (0.02, 0.19)	0.09 (0.0, 0.12)
Tennessee	0.74 (0.62, 0.92)	<0.01 (0.0, 0.0)	0.11 (0.0, 0.15)	0.05 (0.0, 0.07)	0.09 (0.0, 0.12)
Missouri	0.77 (0.67, 0.92)	<0.01 (0.0, 0.0)	0.10 (0.0, 0.13)	0.04 (0.0, 0.06)	0.08 (0.0, 0.11)
Wisconsin	0.83 (0.75, 0.98)	<0.01 (0.0, 0.0)	0.04 (0.0, 0.03)	0.03 (0.0, 0.01)	0.09 (0.0, 0.13)
Louisiana	0.78 (0.67, 0.94)	<0.01 (0.0, 0.0)	0.08 (0.0, 0.11)	0.05 (0.0, 0.08)	0.08 (0.0, 0.11)
South Carolina	0.77 (0.66, 0.93)	<0.01 (0.0, 0.0)	0.10 (0.0, 0.13)	0.06 (0.0, 0.08)	0.06 (0.0, 0.08)
Kansas	0.80 (0.7, 0.94)	<0.01 (0.0, 0.0)	0.08 (0.0, 0.11)	0.04 (0.0, 0.06)	0.07 (0.0, 0.1)
Oklahoma	0.79 (0.69, 0.96)	<0.01 (0.0, 0.0)	0.08 (<0.01, 0.11)	0.04 (0.0, 0.06)	0.08 (<0.01, 0.11)
Kentucky	0.77 (0.67, 0.92)	<0.01 (0.0, 0.0)	0.10 (<0.01, 0.12)	0.05 (0.0, 0.07)	0.07 (0.0, 0.1)
Idaho	0.84 (0.76, 0.98)	<0.01 (0.0, 0.0)	0.07 (<0.01, 0.1)	0.02 (0.0, <0.01)	0.06 (0.0, 0.08)
New Mexico	0.86 (0.79, 1.0)	<0.01 (0.0, 0.0)	0.05 (0.0, 0.07)	0.04 (0.0, 0.04)	0.05 (0.0, 0.07)
Iowa	0.78 (0.68, 0.94)	<0.01 (0.0, 0.0)	0.08 (0.0, 0.1)	0.04 (0.0, 0.05)	0.09 (0.0, 0.13)
Alabama	0.73 (0.6, 0.92)	<0.01 (0.0, 0.0)	0.16 (<0.01, 0.22)	0.05 (<0.01, 0.07)	0.06 (<0.01, 0.08)
Maine	0.77 (0.68, 0.91)	0.02 (0.0, <0.01)	0.07 (0.02, 0.08)	0.07 (0.02, 0.09)	0.08 (<0.01, 0.09)
Alaska	0.78 (0.67, 0.94)	<0.01 (0.0, 0.0)	0.12 (0.0, 0.17)	0.02 (0.0, 0.0)	0.08 (0.0, 0.12)
Nebraska	0.81 (0.72, 0.94)	<0.01 (0.0, 0.0)	0.06 (0.0, 0.07)	0.04 (0.0, 0.05)	0.09 (<0.01, 0.14)
Rhode Island	0.87 (0.8, 1.0)	<0.01 (0.0, 0.0)	0.02 (0.0, 0.0)	0.06 (0.0, 0.08)	0.05 (0.0, 0.07)
Montana	0.90 (0.86, 1.0)	<0.01 (0.0, 0.0)	0.01 (0.0, <0.01)	0.02 (0.0, <0.01)	0.07 (0.0, 0.1)
Arkansas	0.83 (0.75, 0.97)	<0.01 (0.0, 0.0)	0.06 (0.0, 0.08)	0.05 (0.0, 0.07)	0.06 (0.0, 0.09)
Delaware	0.73 (0.63, 0.87)	<0.01 (0.0, 0.0)	0.05 (0.01, 0.07)	0.10 (0.03, 0.14)	0.11 (0.03, 0.15)
Mississippi	0.83 (0.75, 0.96)	<0.01 (0.0, 0.0)	0.05 (0.0, 0.04)	0.06 (0.0, 0.08)	0.07 (0.0, 0.09)
Vermont	0.87 (0.8, 1.0)	<0.01 (0.0, 0.0)	0.05 (0.0, 0.06)	0.03 (0.0, <0.01)	0.05 (0.0, 0.07)
West Virginia	0.85 (0.79, 0.96)	<0.01 (0.0, 0.0)	0.03 (<0.01, 0.03)	0.05 (<0.01, 0.05)	0.07 (<0.01, 0.08)
Wyoming	0.83 (0.75, 0.98)	<0.01 (0.0, 0.0)	0.07 (<0.01, 0.09)	0.02 (<0.01, <0.01)	0.07 (<0.01, 0.09)
North Dakota	0.91 (0.86, 1.0)	<0.01 (0.0, 0.0)	<0.01 (0.0, 0.0)	0.01 (0.0, 0.0)	0.08 (0.0, 0.11)
South Dakota	0.88 (0.82, 1.0)	<0.01 (0.0, 0.0)	<0.01 (0.0, 0.0)	0.02 (0.0, 0.0)	0.09 (0.0, 0.13)

Table S7: Importation of seeding events. Sources are listed from the second column on. Targets are the US states listed in the first column. Numbers are rounded to the second digit.

clockwise in the plot thus looking at countries where the local outbreaks began in February. The range of seeding shares goes from 10% [IQR 0% – 20%] in Italy to 87% [IQR 79% – 100%] in Slovak Republic. With the exceptions of the Netherlands (39% [IQR 20% – 57%]), Finland (37% [IQR 20% – 53%]), Austria (34% [IQR 14% – 50%]), Denmark (33% [IQR 14% – 50%]), Switzerland (32% [IQR 12% – 50%]), and Sweden (32% [IQR 15% – 50%]), all countries that experience a local onset of transmission after the first week of February are characterized by a share of European importations above or equal to 40%.

In Table S7 we report the seeding share for the US states. Within the US, while importations from mainland China contribute to early introductions of the virus, we find that other potential sources of importation play a key role in seeding the epidemic in different places. As shown in Fig. S12-A, the share of infection importations originating from Europe in California was nine times smaller than those in New York state (9% [IQR 0% – 14%]). Among the states for which the model estimates an early onset of local transmission before the third week of February (considering median values), European sources are statistically contributing 12% [IQR 0% – 17%] of SARS-CoV-2 importations for New Jersey, 11% [IQR 0% – 17%] for Florida, and only 4% [IQR 0% – 6%] for Texas. It is important to notice how, for countries in Europe, the US implemented additional travel advisories and restrictions a month later at the end February and early March. The share of importations from Asia is more significant for countries among the first to experience local outbreaks and becomes progressively smaller as we move clockwise in the plot. The range goes from 25% [IQR 10% – 37%] in New Jersey and 21% [IQR 0% – 33%] in New York to values smaller than 1% in North and South Dakota. As we mentioned above, the contribution from Asia is overall smaller than that of Europe. Interestingly, the domestic importations are, across the board, statistically relevant in seeding the epidemic in many states. Among the states for which we estimated a late onset of local transmission (second half of February), domestic sources account for 81% [IQR 72% – 94%] of the virus introductions in Nebraska, 86% [IQR 79% – 100%] in New Mexico, 83% [IQR 75% – 97%] in Arkansas, and 91% [IQR 86% – 100%] in North Dakota.

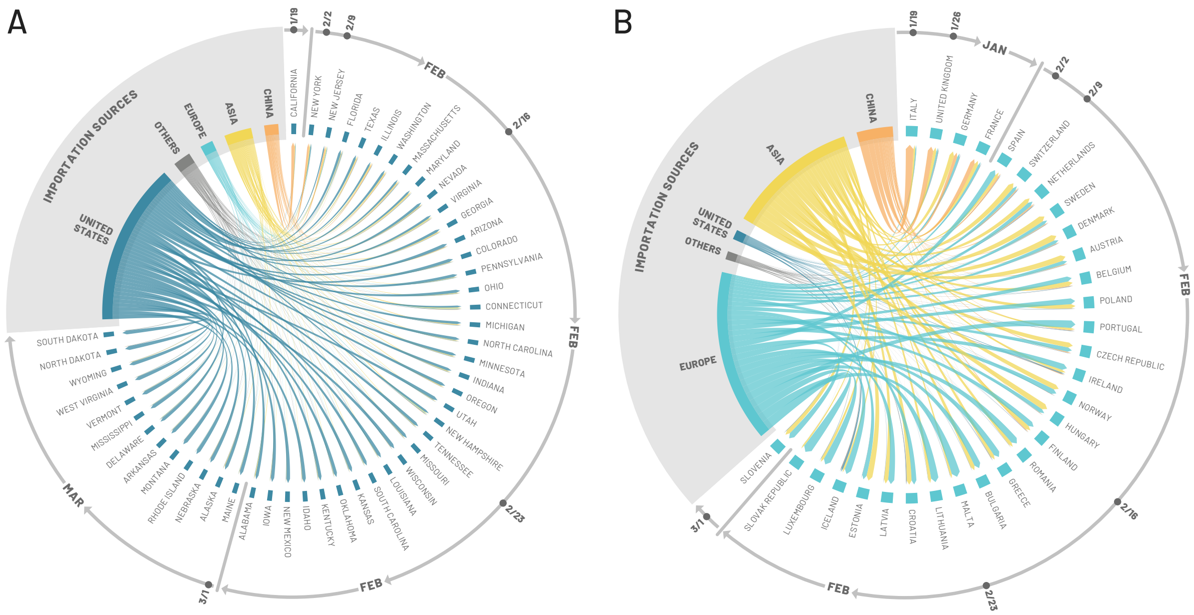

Figure S12: Share of importations of infections in all continental states (A) and in European countries (C) from US, China, Europe, Asia and all other countries before the start of the local outbreak. US states and European countries are ordered, clockwise, according to the start of the local outbreak.

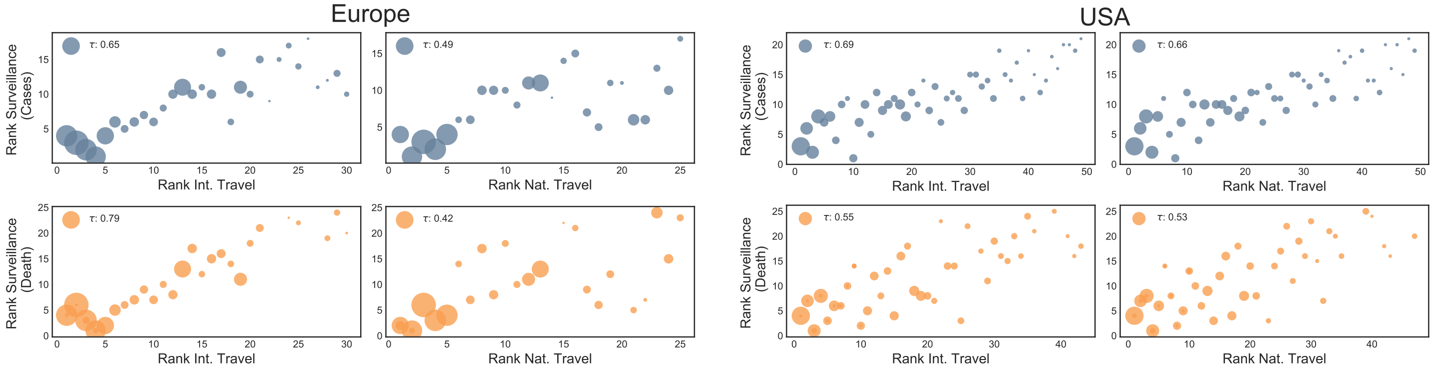

Figure S13: Correlation between the order in which states reached 100 confirmed cases (top row) or deaths (bottom row) and their International (left) or National (right) air traffic. The size of each state is assigned proportional to the population size. The first two columns refer to countries in Europe. The last two to the US states. For the US we consider n = 49 and for Europe n = 30.

6 Correlation Analysis

As mentioned and shown in the main text, during the early phases of the spreading, mobility plays a crucial role. In order to highlight this aspect, here we report the full correlation analysis between the real data and mobility indicators. In particular, we compute the order in which states reached 100 cases/deaths in the surveillance data and compare it with the order of European countries and US states according to their air traffic (considering both national and international travels). Note how the correlation plot in the main text considered as a mobility indicator the sum of the two types of traffic. In Fig. S13 we show the result reporting also the value of the Kendall's tau. In European countries both cases and deaths are highly correlated with international travels. The national flows are less correlated. In US states, the rank of cases are more highly correlated with both international and national travels than deaths.

The countries and states that were the first to experience the outbreak, besides being hubs in the air transportation network, are also very populous. It is then natural to wonder how rankings based on population compare with respect to those based on air traffic. In Figure S14 we show the comparison. In particular, we order European countries and US states according to their population size and density and to the epidemic indicators from surveillance (cases and deaths). We find high correlation levels with population ranks for both Europe and US states for both cases and deaths. It is interesting to note that the correlations with air travel are either comparable or stronger than those with population or density. The correlation for the number of deaths (bottom row) is lower with respect to the number of cases (top row) for US. Furthermore, the correlations are even smaller when considering population density, especially in the case of cases in Europe.

In Figure S15 we repeat the same analysis considering the model's projections. The correlations are comparable to the previous analysis. Similarly, model projections are more highly correlated with population sizes and less correlated with population density.

It is important to observe that air travel traffic, population and population densities are not independent indicators. Figure S16 highlights this observation. Particularly high is the correlation between air traffic and population. Also, population and population densities are well correlated (especially in the US) while air traffic and density are not. This is due, in part, to the fact that many countries or states experience a high volume of air traffic due to their geographic location, but are not particularly densely populated.

The correlations between rankings reported above have been computed by using the Kendall's tau (47) as implemented by the *scipy.stats* library (48). The metric is designed to compare the rankings obtained ordering items, states in our case, according to pairs of different quantities. The Kendall's tau is defined

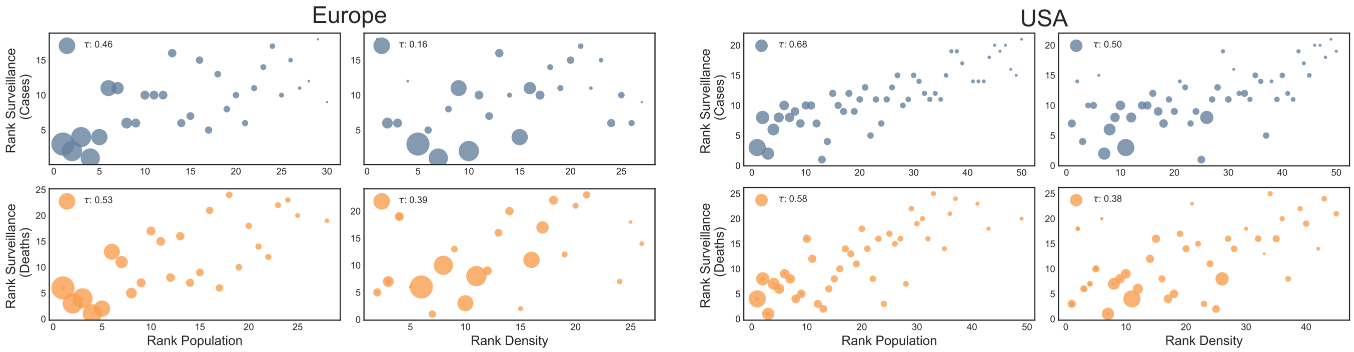

Figure S14: Correlation between the order in which states reached 100 confirmed cases (top row) or deaths (bottom row) and their population (left) or population density (right). The size of each state is assigned proportional to the population size. The first two columns refer to countries in Europe and the last two the US states. For the US we consider n = 49 and for Europe n = 30.

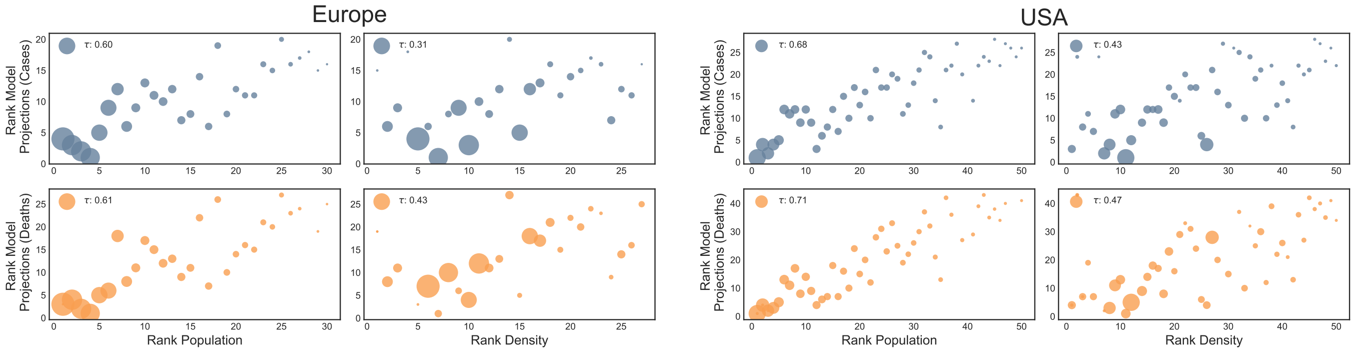

Figure S15: Correlation between the order in which states reached 100 confirmed cases (top row) or deaths (bottom row) according to the model and their population or population density. On the left we consider the case of European countries while on the right US states. The size of each country/state is assigned proportional to the population size. For the US we consider n = 49 and for Europe n = 30.

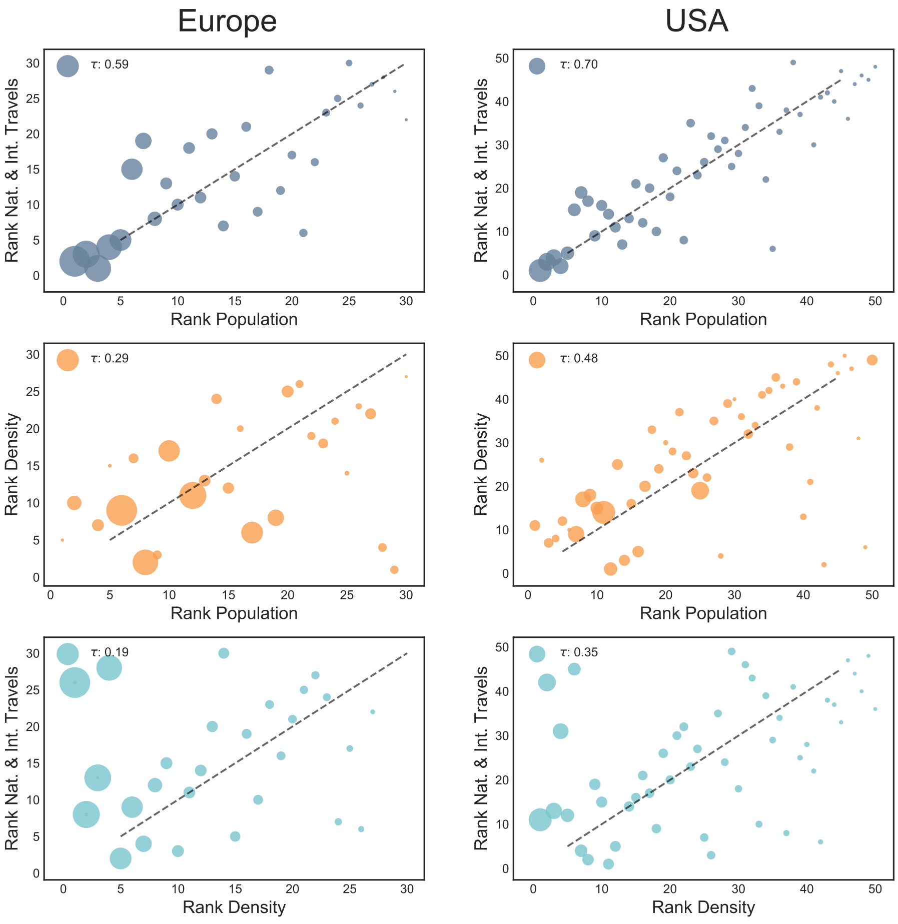

Figure S16: Correlation between travel, population and population density for the European countries (left) and continental US states (right). Circle size corresponds to the population of each state and country. For the US we consider n = 49 and for Europe n = 30.

only in the case that the ranks have the same size. In case the two ranks have different size (i.e., some states did not yet go above a given threshold) the metric is applied to the common subset of the two.

7 Analysis of additional countries in different regions of the world

Although the presented analysis focuses on the US and European countries, the model is global hence it generates data for a large number of countries and territories in different regions of the world. Many countries and regions experienced an "initial wave" during different time windows relative to those considered in the US and Europe. Analysis of pandemic trajectories in each country would require a detailed, case by case investigation. Nevertheless, here we provide an analysis of the first 6-8 months of the COVID-19 pandemic for 24 additional countries: Argentina, Australia, Canada, Chile, Colombia, Egypt, Ethiopia, India, Indonesia, Japan, Kenya, Korea, Rep., Malaysia, Mexico, New Zealand, Nigeria, Philippines, Singapore, South Africa, Sudan, Taiwan, Tunisia, Turkey, and the United Arab Emirates. Countries were selected if they had at least 100 deaths by July 4, 2020, with the exception of a couple countries in Asia, Oceania, and Africa in order to be globally representative. Our results convey that the large heterogeneity of the pandemic is due to the interplay of the global mobility network, travel restriction policies, and NPIs such as social distancing guidelines and strong contact-tracing and testing efforts.

7.1 Onset of local transmission. In Fig. S17, we show the posterior distributions of the onset of local transmission for 24 additional countries investigated. The definition of the onset of local transmission for a country is the earliest date when at least 10 new infections are generated per day. The methodology is the same as that of the main text, which was used to analyze the onset of local transmission in Europe and the US. The figure readily shows that the onset of local transmission is heterogeneous across countries and it could differ on the order of a month. As expected, we observe earlier epidemic take off times in countries such as Japan, Korea, Rep., and Malaysia, while some African and Latin American countries have a median value of the onset of local transmission a few weeks later. Furthermore, the posterior distributions are wide and the support of each distribution spans several weeks. It is worth remarking that an early local onset is not necessarily related to an initial, large epidemic wave in countries who adopted quick and aggressive mitigation measures and rigid travel restrictions. In Fig. S17 B) we report the correlation between the model estimated dates of the onset of local transmission (median values from Fig. S17 (A)) and date where at least 50 cases were reported in that country (B, left) and the date of the first reported death (B, right). We find a strong correlation between the model estimated onset of local transmission and the two surveillance based metrics. However, as expected, there is a delay of a couple weeks due in part to the testing capacity and contact tracing efforts.

7.2 COVID-19 burden. Here we estimate the disease burden in the 24 selected countries across the world over the first 6-8 months of the COVID-19 pandemic. Within each country, the pandemic trajectory is driven by the establishment and timing of NPIs, travel restrictions, and the epidemiological relevant features (i.e., population size and density, age-structure etc.) which are spatially heterogeneous. We use the ABC rejection approach described in Section 3.2 to estimate the posterior distribution of the infection AR and IFR for each new country analyzed.

In Fig. S18 we report the model fit of the estimated weekly deaths and in Fig. S19 we report the posterior distribution of the infection AR and the IFR for the 24 additional countries in different regions of the world. It is worth remarking that for countries outside the US and Europe it is not always possible to identify a clear first wave in the time window analyzed. While countries like Australia, Canada, and Japan experienced both a growth and decline in observed deaths during the time period shown, other countries in Latin America and Africa such as Argentina, Colombia, and Egypt are at different stages of the pandemic. The mitigation strategies and intervention policies implemented in each country give rise to very different epidemic trajectories, both in magnitude and timing. As mentioned in the previous section, the epidemic evolution is not determined by the onset of local transmission, but rather is driven by the timing and implementation of the NPIs in each specific location. It is also interesting to notice that the estimated median IFR across countries appears to be more broadly distributed than what we observe in the US and Europe. Indeed, while Europe and the US have a relatively similar population

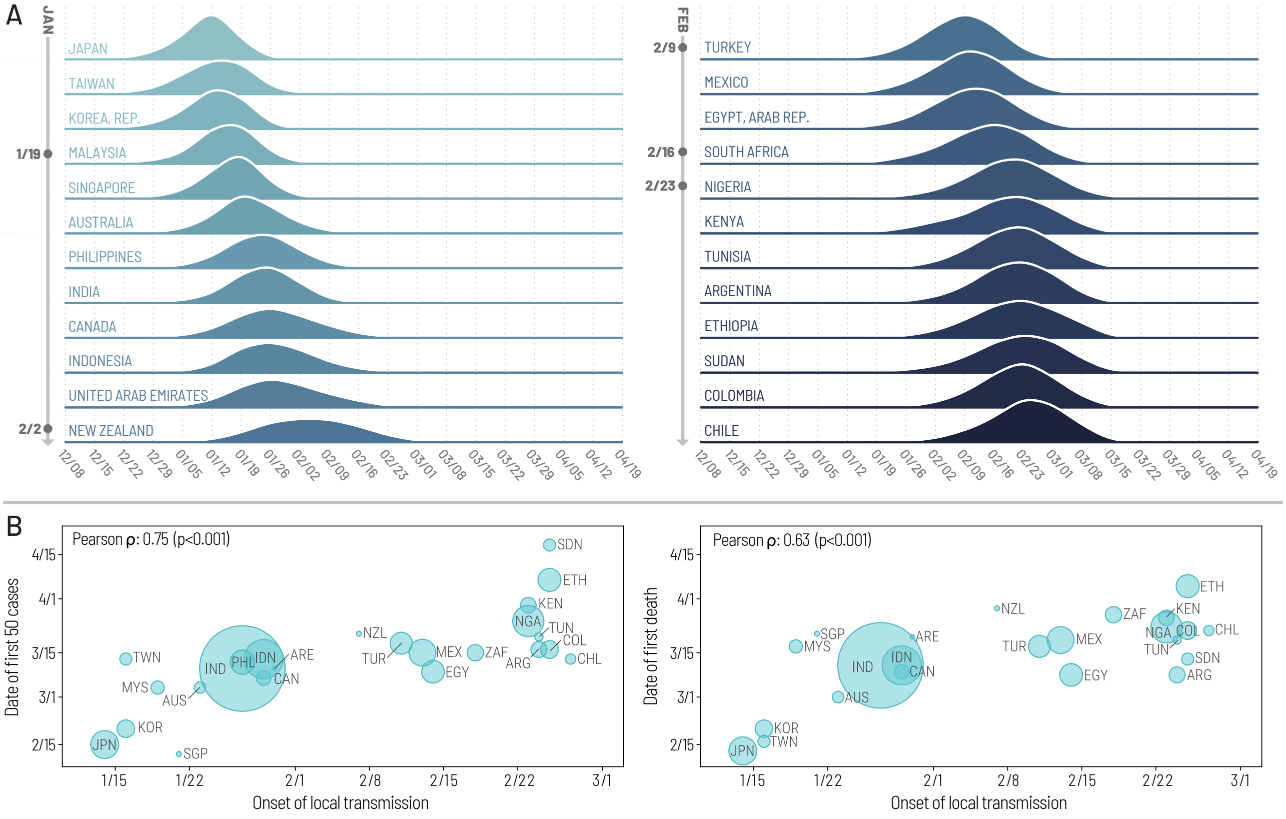

Figure S17: (A) The posterior distribution of the model estimated onset of local transmission for 24 countries in Asia, Oceania, North and South America, and Africa. (B, left) The correlation (n = 24) between the model estimates of the dates of the onset of local transmission and the date where at least 50 cumulative cases were reported. (B, right) The correlation (n = 24) between the dates of the model estimates of the onset of local transmission and the date of the first reported death. The size of the circles in B correspond to the population size of each country and the surveillance data used is taken from Ref. (49). The correlations are calculated using the Pearson correlation coefficient ρ.

demographic structure, several low and middle income countries have overall young populations. Since the age stratified IFR is extremely skewed towards the older age brackets, countries with generally young populations exhibit low overall IFRs, even in the presence of considerable infection ARs. It is worth noting that a full discussion of the differences across world regions deserves a longer discussion around the different mitigation approaches as well as socio-demographic characteristics.

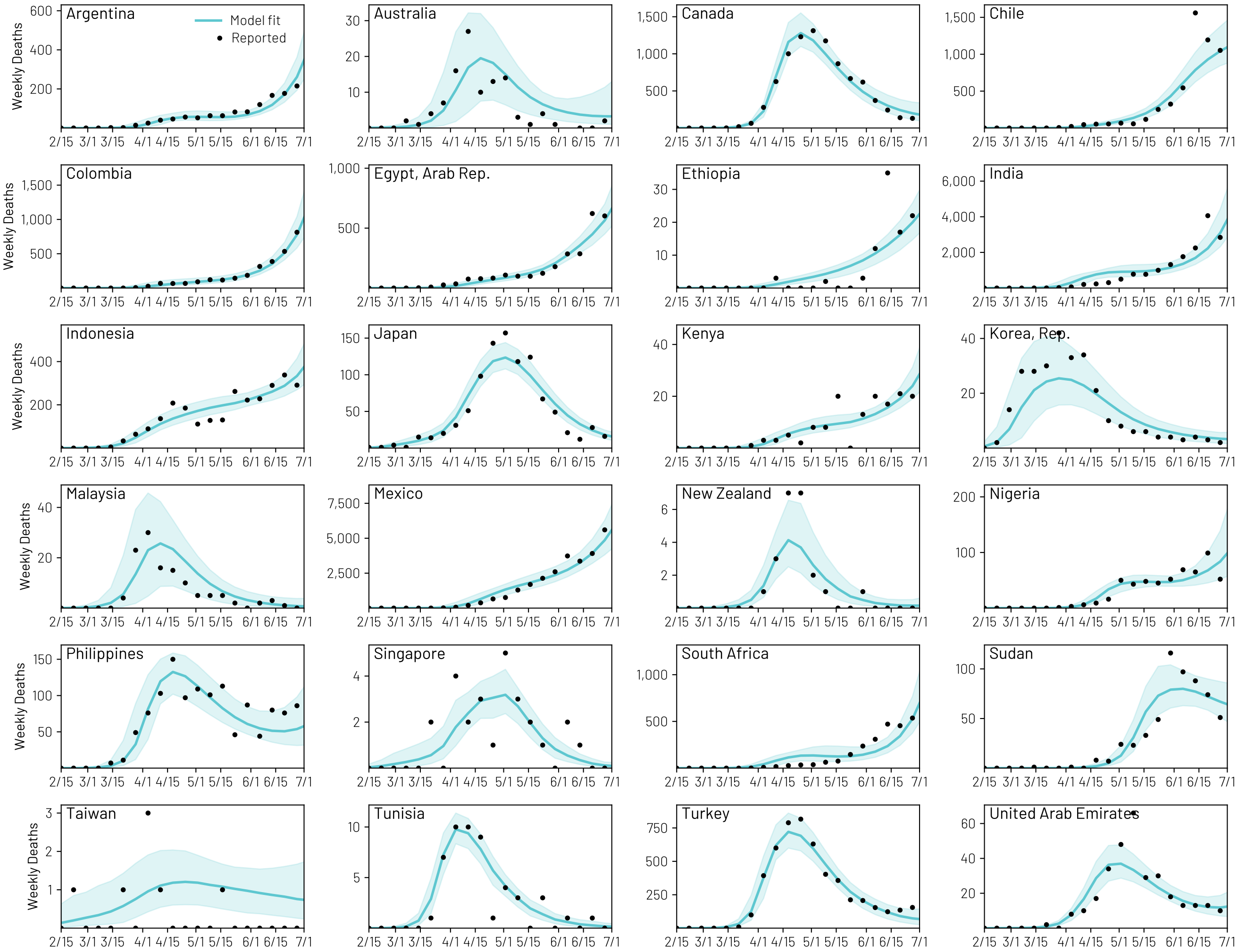

Figure S18: Model fits of the weekly deaths for 24 countries outside of the US and Europe. We report the median value and the 90% CI.

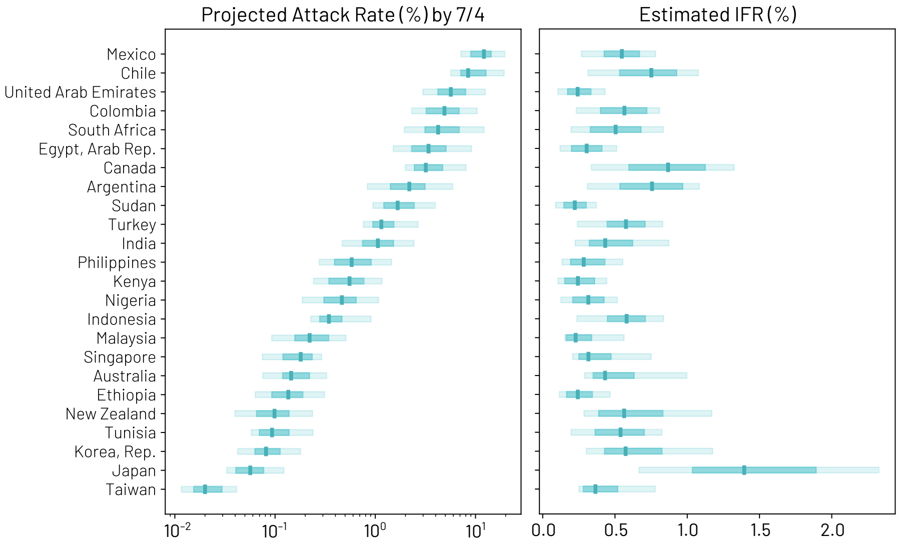

Figure S19: The estimated infection ARs and IFRs by July 4, 2020, for a subset of global countries. These posterior distributions are the result of the ABC analysis of 200,000 independent model realizations. The outer, lighter boxes represent the 90%CI, the darker, inner boxes represent the IQR, and the vertical lines represent the median values.

8 Counterfactual scenarios for testing and infection detection

Overall, the results presented in the main text of this work show that testing based on travel history from countries with established COVID-19 local transmission, were not able to prevent the dispersion of undetected cases that seeded the global spread of the epidemic. In order to provide a numerical estimate of the benefit offered by broader testing specifications, we generate a counterfactual scenario where the surveillance systems of the US states and European countries are imagined to operate at levels able to identify 50% of all imported and locally generated infections. We find that while this is an optimistic counterfactual, it clearly indicates that broadening the testing requirements beyond travel history and to countries known to have local transmission can truly impede the pandemic. In Fig. S20 we report the probability each country generated more than 10 cumulative infections per 1 million people as a function of time in a few countries that experienced early local transmission. Specifically, the probability is defined as the fraction of stochastic runs that cross the population dependent threshold on the specific day. We see in the baseline scenario (the model used in the main text) that while the probability of having 10 cumulative infections per million inhabitants crosses the threshold of 90% by the end of February in all the reported countries, within the counterfactual scenarios, no country crosses that threshold before the end of March. The threshold of 10 infections per million corresponds in most countries to detection of a few hundred infections, however this task should not be underestimated as, especially during the respiratory illness season, it implies a considerably large number of tests based on etiological indications and travel histories, as well as the careful contact tracing of all detected infections. However the results clearly show that broadening testing specifications could have considerably slowed the pandemic progression, buying considerable time to prepare mitigation responses.

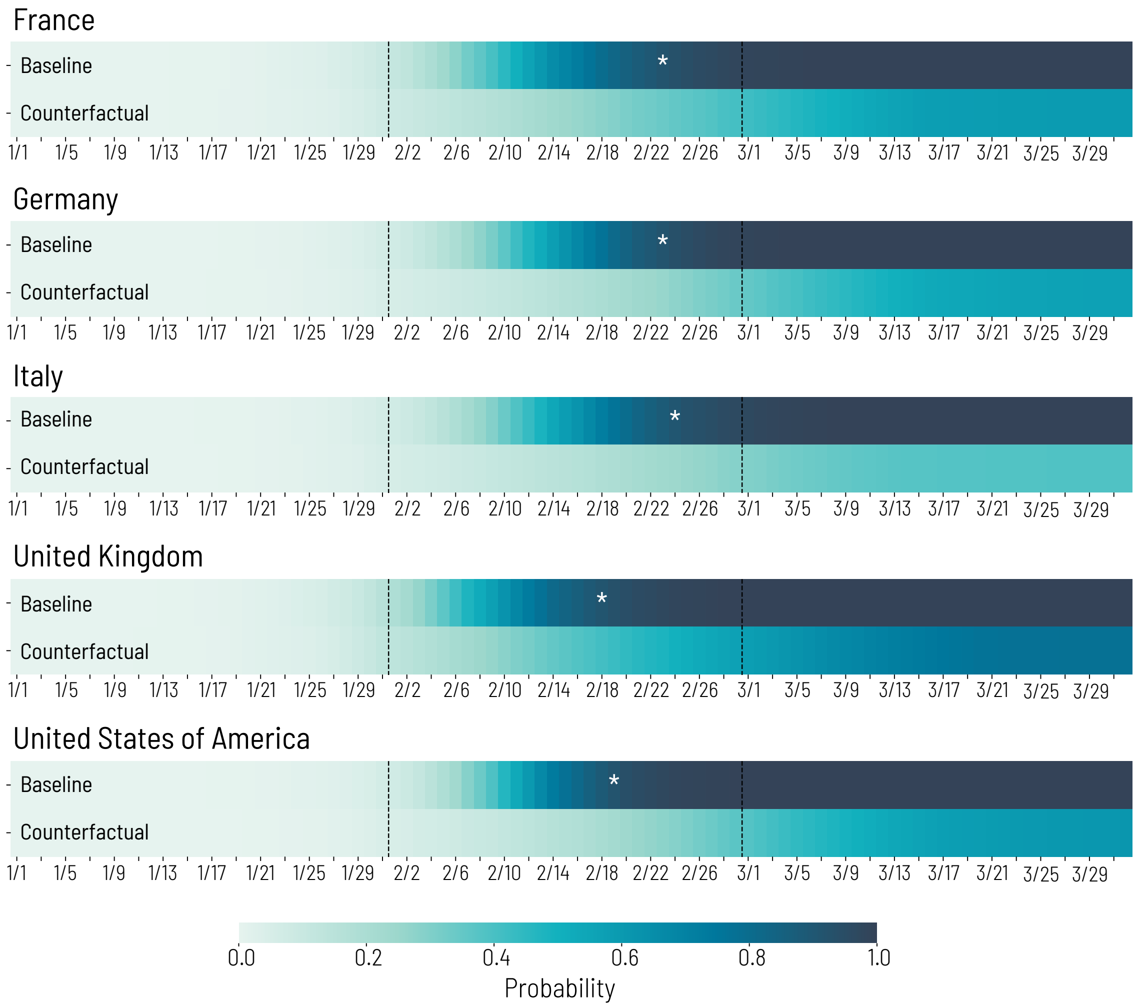

Figure S20: The probability that a country observes 10 cumulative infections per 1 million inhabitants by a particular date. The white star represents the date where the probability is greater than 90%. We compare the baseline model estimates reported in the main text (see Section 2) to the counterfactual scenario described in Section 8.

Country	Age	Study period	Survey (%)	Model (median, %)	Model (90%CI, %)	Ref.
Denmark	17-69	04/27/20-05/03/20	1.9 [95% CI 0.8-2.3]	**1.05**	[0.71-2.71]	(51)
France	0+	05/11/20-05/17/20	4.9 [CI N/A]	**4.60**	[3.34-9.95]	(52)
Czech Republic	18-89	04/23/20-05/01/20	0.4 [CI N/A]	**0.38**	[0.24-0.95]	(52)
Portugal	1+	05/21/20-07/08/20	2.7 [CI N/A]	**1.60**	[0.94-4.82]	(52)
Sweden	0-95	06/08/20-06/14/20	5.6 [CI N/A]	**6.26**	[3.72-14.80]	(52)
Hungary	14+	05/01/20-05/16/20	0.68 [CI N/A]	**0.71**	[0.43-1.80]	(53)
Spain	0+	04/27/20-05/11/20	4.6 [95% CI 4.3-5.0]	**7.12**	[5.11-14.44]	(54)
Italy	0+	05/25/20-07/15/20	2.6 [CI N/A]	**4.97**	[3.21-12.78]	(55)
Netherlands	18-72	05/11/20-05/18/20	5.6 [CI N/A]	**4.67**	[2.93-11.34]	(52)
Belgium		05/10/20	8.4 [95% CI 6.6-11]	**12.79**	[8.24-27.16]	(56)
United Kingdom		05/24/20	6.78 [95% CI 5.21-8.64]	**6.44**	[4.13-14.37]	(57)
**State/City**	**Age**	**Study period**	**Survey (%)**	**Model (median, %)**	**Model (90%CI, %)**	**Ref.**
Los Angeles, CA		04/10/20-04/11/20	4.1 [CI 2.8-5.6]	**0.89**	[0.31-2.48]	(58)
Connecticut	0-65+	04/26/20-05/03/20	4.94 [95% CI 3.61 – 6.52]	**8.11**	[5.91-17.60]	(50)
Louisiana	0-65+	04/01/20-04/08/20	5.75 [95% CI 3.87 – 8.23]	**3.92**	[2.68-10.01]	(50)
Minneapolis, MN	0-65+	04/30/20-05/12/20	2.35 [95% CI 0.98 – 4.50]	**3.87**	[2.51-9.81]	(50)
Missouri	0-65+	04/20/20-04/26/20	2.65 [95% CI 1.65-3.86]	**1.43**	[0.96-3.60]	(50)
Philadelphia, PA	0-65+	04/13/20-04/25/20	3.19 [95% CI 1.69 – 5.19]	**6.26**	[2.18-13.74]	(50)
San Francisco Bay Area, CA	0-65+	04/23/20-04/27/20	0.97 [95% CI 0.34 – 2.44]	**1.97**	[0.51-6.54]	(50)
New York	18+	04/19/20-04/28/20	14.0 [95% CI 13.3–14.7]	**12.98**	[8.72-26.20]	(59)
New York City	18+	04/19/20-04/28/20	22.7 [95% CI 21.5–24.0]	**19.78**	[12.78-38.68]	(59)

Table S8: Summary of the country/state/city level serological studies used for comparison against model estimates. Each row refers to a country, state, or city and the columns include information about the serology study such as the age range of participants, the study period, and the estimated prevalence of SARS-CoV-2 (Survey). Confidence intervals are provided if they are found in the reference. For each region we report the model estimates of the infection AR as of the last day of the study period (median value and 90%CI). The data in this table are visualized in Extended Data Fig. 1 D.

9 Data

9.1 Epidemic surveillance data. The surveillance data of the reported cases and deaths are taken from the John Hopkins University Coronavirus Resource Center (49).

9.2 Model intervention data. The model incorporates Google COVID-19 Community Mobility Reports data (24) to estimate, on the one hand, changes in local mobility and, on the other hand, changes in contact patterns in workplaces and in the general community. NPIs and other policy interventions are tracked using the Oxford Covid-19 Government Response Tracker (OxCGRT) (22). Lastly, reductions in air travel are computed by considering the percent change between the monthly origin-destination passenger flows between corresponding months in 2020 and 2019 (7). Implementation details are provided in Section 1.2.

9.3 Serological data comparison. We did an extensive literature search for serological studies performed from April-July 2020. In Extended Data Figure 1D, in the main text, we show the correlation between the estimated prevalence of SARS-CoV-2 antibodies and the model's estimated infection AR reported on the last date of that study. In Table S8 we report the prevalence values and study date ranges for each serological survey considered along with our estimated infection AR. The US cities Minneapolis, Philadelphia, and the San Francisco Bay Area refer to metropolitan areas or combined statistical area containing that city (for specifics on the location of the testing sites see Ref. (50)).

References

[1] Balcan, D. *et al.* Modeling the spatial spread of infectious diseases: The GLobal Epidemic and Mobility computational model. *J. Comput. Sci.* **1**, 132–145 (2010).

[2] Balcan, D. *et al.* Multiscale mobility networks and the spatial spreading of infectious diseases. *Proc. Natl Acad. Sci. USA* **106**, 21484–21489 (2009).

[3] Socioeconomic Data and Applications Center (SEDAC), Columbia University `http://sedac.ciesin.columbia.edu/gpw`.

[4] Mistry, D. *et al.* Inferring high-resolution human mixing patterns for disease modeling. *Nat. Commun* **12**, 1–12 (2021).

[5] Prem, K., Cook, A. R. & Jit, M. Projecting social contact matrices in 152 countries using contact surveys and demographic data. *PLoS Comput. Biol.* **13**, e1005697 (2017).

[6] International Air Transportation Association; `https://www.iata.org/`.

[7] Official Aviation Guide; `https://www.oag.com/`.

[8] Simini, F., González, M. C., Maritan, A. & Barabási, A.-L. A universal model for mobility and migration patterns. *Nature* **484**, 96–100 (2012). `https://doi.org/10.1038/nature10856`.

[9] Verity, R. *et al.* Estimates of the severity of coronavirus disease 2019: a model-based analysis. *Lancet Infect Dis* (2020). `https://doi.org/10.1016/S1473-3099(20)30243-7`.

[10] Backer, J. A., Klinkenberg, D. & Wallinga, J. Incubation period of 2019 novel coronavirus (2019-nCoV) infections among travellers from Wuhan, China, 20–28 January 2020. *Euro Surveill.* **25**, 2000062 (2020).

[11] Kissler, S. M., Tedijanto, C., Goldstein, E., Grad, Y. H. & Lipsitch, M. Projecting the transmission dynamics of SARS-CoV-2 through the postpandemic period. *Science* **368**, 860–868 (2020). `https://science.sciencemag.org/content/368/6493/860`.

[12] Li, Q. *et al.* Early transmission dynamics in Wuhan, China, of novel coronavirus–infected pneumonia. *N. Engl. J. Med.* **579**, 1199-1207 (2020).

[13] Griffin, J. *et al.* Rapid review of available evidence on the serial interval and generation time of COVID-19. *BMJ Open* **10** (2020). `https://bmjopen.bmj.com/content/10/11/e040263`.

[14] Chinazzi, M. *et al.* The effect of travel restrictions on the spread of the 2019 novel coronavirus (COVID-19) outbreak. *Science* **368**, 395–400 (2020).

[15] Baidu Qianxi. `http://qianxi.baidu.com/` (2020).

[16] New York Times. North Korea Bans Foreign Tourists Over Coronavirus, Tour Operator Says. `https://www.nytimes.com/2020/01/21/world/asia/coronavirus-china-north-korea-tourism-ban.html` (2020).

[17] CNA. Scoot cancels flights to China's Wuhan over virus outbreak. `https://www.channelnewsasia.com/news/singapore/wuhan-virus-scoot-cancels-flights-mtr-train-12309076` (2020).

[18] Toui tre News. Vietnam aviation authority ceases all flights to and from coronavirus-stricken Wuhan. `https://tuoitrenews.vn/news/business/20200124/vietnam-aviation-authority-ceases-all-flights-to-and-from-coronavirusstricken-wuhan/52707.html` (2020).

[19] Reuters. Russia ramps up controls, shuts China border crossings over virus fears. https://www.reuters.com/article/us-china-health-russia-border/russian-regions-in-far-east-close-border-with-china-amid-coronavirus-fears-tass-idUSKBN1ZR0TU (2020).

[20] Center for Disease Control. Novel Coronavirus in China. https://wwwnc.cdc.gov/travel/notices/warning/novel-coronavirus-china (2020).

[21] The Australian. "Travelers from China to be denied entry to Australia. https://www.theaustralian.com.au/subscribe/news/1/?sourceCode=TAWEB_WRE170_a&dest=https%3A%2F%2Fwww.theaustralian.com.au%2Fnation%2Ftravellers-from-china-to-be-denied-entry-into-australia%2Fnews-story%2F7b7619d44af78dd7395a934e22b52997&memtype=anonymous&mode=premium (2020).

[22] Hale, T., Webster, S., Petherick, A., Phillips, T. & Kira, B. Oxford COVID-19 Government Response Tracker. Blavatnik School of Government (2020).

[23] Zhang, J. *et al.* Changes in contact patterns shape the dynamics of the COVID-19 outbreak in China. *Science* **368**, 1481-1486, (2020). https://science.sciencemag.org/content/early/2020/04/28/science.abb8001. https://science.sciencemag.org/content/early/2020/04/28/science.abb8001.full.pdf.

[24] Google LLC. "Google COVID-19 Community Mobility Reports". https://www.google.com/covid19/mobility/.

[25] De Luca, G. *et al.* The impact of regular school closure on seasonal influenza epidemics: a data-driven spatial transmission model for Belgium. *BMC infectious diseases* **18**, 1–16 (2018).

[26] van Dorp, L. *et al.* Emergence of genomic diversity and recurrent mutations in SARS-CoV-2. *Infection, Genetics and Evolution* **83**, 104351 (2020). https://www.sciencedirect.com/science/article/pii/S1567134820301829.

[27] A. Rambaut, "Preliminary phylogenetic analysis of 11 nCoV2019 genomes, 2020-01-19" (2020). http://virological.org/t/preliminary-phylogenetic-analysis-of-11-ncov2019-genomes-2020-01-19/329.

[28] Imai, Natsuko and Cori, Anne and Dorigatti, Ilaria and Baguelin, Marc and Donnelly, Christl A. and Riley, Steven and Ferguson, Neil M. Report 3: Transmissibility of 2019-nCoV . https://www.imperial.ac.uk/mrc-global-infectious-disease-analysis/covid-19/report-3-transmissibility-of-covid-19/ (2020).

[29] K. Anderson, "Clock and TMRCA based on 27 genomes"(2020); http://virological.org/t/clock-and-tmrca-based-on-27- genomes/347.

[30] T. Bedford et al., "Genomic analysis of nCoV spread. Situation report 2020-01-23" (2020); https://nextstrain.org/narratives/ ncov/sit-rep/2020-01-23.

[31] De Salazar, P. M., Niehus, R., Taylor, A., Buckee, C. & Lipsitch, M. Identifying Locations with Possible Undetected Imported Severe Acute Respiratory Syndrome Coronavirus 2 Cases by Using Importation Predictions. *Emerging Infectious Diseases* **26** (2020).

[32] Gostic, K., Gomez, A. C., Mummah, R. O., Kucharski, A. J. & Lloyd-Smith, J. O. Estimated effectiveness of symptom and risk screening to prevent the spread of COVID-19. *Elife* **9**, e55570 (2020).

[33] Niehus, R., De Salazar, P. M., Taylor, A. R. & Lipsitch, M. Using observational data to quantify bias of traveller-derived COVID-19 prevalence estimates in Wuhan, China. *The Lancet Infectious Diseases* **20**, 803–808 (2020).

[34] Global security index. https://www.ghsindex.org/.

[35] Oran, D. P. & Topol, E. J. Prevalence of Asymptomatic SARS-CoV-2 Infection. *Annals of Internal Medicine* **173**, 362–367 (2020). https://doi.org/10.7326/M20-3012.

[36] COVID-19 Pandemic Planning Scenarios; https://www.cdc.gov/coronavirus/2019-ncov/hcp/planning-scenarios.html.

[37] Cordasco, G. & Gargano, L. Community detection via semi-synchronous label propagation algorithms. In *2010 IEEE International Workshop on: Business Applications of Social Network Analysis (BASNA)*, 1–8 (IEEE, 2010).

[38] Reuters. "Croatia confirms its first case of coronavirus infection". https://www.reuters.com/article/us-croatia-coronavirus/croatia-confirms-its-first-case-of-coronavirus-infection-idUSKBN20J1OB (2020).

[39] Beaumont, M. A., Zhang, W. & Balding, D. J. Approximate Bayesian Computation in Population Genetics. *Genetics* **162**, 2025–2035 (2002). https://www.genetics.org/content/162/4/2025. Publisher: Genetics _eprint: https://www.genetics.org/content/162/4/2025.full.pdf.

[40] Althouse, B. M. *et al.* Superspreading events in the transmission dynamics of sars-cov-2: Opportunities for interventions and control. *PLOS Biology* **18**, 1–13 (2020). https://doi.org/10.1371/journal.pbio.3000897.

[41] Sun, K. *et al.* Transmission heterogeneities, kinetics, and controllability of SARS-CoV-2. *Science* **371**, eabe2424 (2021). https://www.science.org/doi/10.1126/science.abe2424. Publisher: American Association for the Advancement of Science.

[42] Bi, Q. *et al.* Epidemiology and transmission of COVID-19 in 391 cases and 1286 of their close contacts in Shenzhen, China: a retrospective cohort study. *The Lancet Infectious Diseases* **20**, 911-919 (2020). https://doi.org/10.1016/S1473-3099(20)30287-5.

[43] Wang, L. *et al.* Inference of person-to-person transmission of COVID-19 reveals hidden super-spreading events during the early outbreak phase. *Nature Communications* **11**, 5006 (2020). https://www.nature.com/articles/s41467-020-18836-4. Bandiera_abtest: a Cc_license_type: cc_by Cg_type: Nature Research Journals Number: 1 Primary_atype: Research Publisher: Nature Publishing Group Subject_term: Epidemiology;Phylogenomics;SARS-CoV-2;Viral infection Subject_term_id: epidemiology;phylogenomics;sars-cov-2;viral-infection.

[44] Sneppen, K., Nielsen, B. F., Taylor, R. J. & Simonsen, L. Overdispersion in COVID-19 increases the effectiveness of limiting nonrepetitive contacts for transmission control. *Proceedings of the National Academy of Sciences* **118** (2021). https://www.pnas.org/content/118/14/e2016623118. Publisher: National Academy of Sciences _eprint: https://www.pnas.org/content/118/14/e2016623118.full.pdf.

[45] Endo, A., Group, C. f. t. M. M. o. I. D. C.-. W., Abbott, S., Kucharski, A. J. & Funk, S. Estimating the overdispersion in COVID-19 transmission using outbreak sizes outside China. Tech. Rep. 5:67, Wellcome Open Research (2020). https://wellcomeopenresearch.org/articles/5-67. Type: article.

[46] Cross, P. C., Johnson, P. L., Lloyd-Smith, J. O. & Getz, W. M. Utility of ¡i¿r¡/i¿¡sub¿0¡/sub¿ as a predictor of disease invasion in structured populations. *Journal of The Royal Society Interface* **4**, 315–324 (2007). https://royalsocietypublishing.org/doi/abs/10.1098/rsif.2006.0185. https://royalsocietypublishing.org/doi/pdf/10.1098/rsif.2006.0185.

[47] Kendall, M. G. A new measure of rank correlation. *Biometrika* **30**, 81–93 (1938).

[48] Scipy.org: Kendall Tau; https://docs.scipy.org/doc/scipy/reference/generated/scipy.stats.kendalltau.html.

[49] Johns Hopkins University Coronavirus Resource Center. https://coronavirus.jhu.edu/.

[50] Havers, F. P. *et al.* Seroprevalence of antibodies to sars-cov-2 in 10 sites in the united states, march 23-may 12, 2020. *JAMA internal medicine* **180**, 1576–1586 (2020).

[51] Erikstrup, C. *et al.* Estimation of SARS-CoV-2 infection fatality rate by real-time antibody screening of blood donors. *Clinical Infectious Diseases* **72**, 249–253 (2021).

[52] O'Driscoll, M. *et al.* Age-specific mortality and immunity patterns of sars-cov-2. *Nature* **590**, 140–145 (2021).

[53] Merkely, B. *et al.* Novel coronavirus epidemic in the Hungarian population, a cross-sectional nationwide survey to support the exit policy in Hungary. *GeroScience* **42**, 1063–1074 (2020).

[54] Pollán, M. *et al.* Prevalence of sars-cov-2 in spain (ene-covid): a nationwide, population-based seroepidemiological study. *The Lancet* **396**, 535–544 (2020).

[55] ISTAT. "Primi risultati dell'indagine di sieroprevalenza sul SARS-CoV-2", url-https://www.istat.it/it/archivio/246156, (2020).

[56] Russell, T. W. *et al.* Reconstructing the early global dynamics of under-ascertained COVID-19 cases and infections. *BMC medicine* **18**, 1–9 (2020).

[57] Office for National Statistcs, UK. Coronavirus (COVID-19) Infection Survey pilot: 28 May 2020. https://www.ons.gov.uk/peoplepopulationandcommunity/healthandsocialcare/conditionsanddiseases/bulletins/coronaviruscovid19infectionsurveypilot/28may2020 (2020).

[58] USC Annenberg Media. USC Student Health begins testing community for antibodies. https://www.uscannenbergmedia.com/2020/04/20/usc-los-angeles-county-covid-19-antibody-test-results-revealed/ (2020).

[59] Rosenberg, E. S. *et al.* Cumulative incidence and diagnosis of SARS-CoV-2 infection in New York. *Annals of epidemiology* **48**, 23–29 (2020).
